# Supplementary material for: Antiviral metabolite 3′-deoxy-3′,4′-didehydro-cytidine is detectable in serum and identifies acute viral infections including COVID-19
Source: Med. 2022 Mar 11;3(3):204–215.e6. doi: 10.1016/j.medj.2022.01.009 (PMC8801973; doi:10.1016/j.medj.2022.01.009)
Supplement: Document S1. Tables S1–S4 and Figures S1–S4 [file mmc1.pdf]

Med, Volume *3*

Supplemental information

Antiviral metabolite 3′-deoxy-3′,4′-didehydro-cytidine is detectable in serum and identifies acute viral infections including COVID-19

Ravi Mehta, Elena Chekmeneva, Heather Jackson, Caroline Sands, Ewurabena Mills, Dominique Arancon, Ho Kwong Li, Paul Arkell, Timothy M. Rawson, Robert Hammond, Maisarah Amran, Anna Haber, Graham S. Cooke, Mahdad Noursadeghi, Myrsini Kaforou, Matthew R. Lewis, Zoltan Takats, and Shiranee Sriskandan

Antiviral metabolite 3'-Deoxy-3',4'-didehydro-cytidine is detectable in serum and identifies acute viral infections including COVID-19

Supplementary Information

Supplementary Table 1 (related to Figure 1). Patient demographics and sample numbers in discovery and validation cohorts. 2

Supplementary Table 2 (related to Figure 1). Confirmed pathogens in discovery and validation cohorts. 3

Supplementary Table 3 (related to Figure 2). Cross-validation of feature performance using FS-PLS. 4

Supplementary Table 4 (related to Figure 4). Five gene transcipts most highly correlated with ddhC in the discovery primary analysis cohort. 5

Supplementary Figure 1 (related to Figure 2). Discovery cohort metabolomic findings and discovery of ddhC. 6

Supplementary Figure 2 (related to Figure 2). Discovery cohort AUCs and relative feature intensity data for all samples and influence of host demographic and clinical factors on feature intensity. 8

Supplementary Figure 3 (related to Figure 3). Validation cohort metabolomic findings and severity analysis. 10

Supplementary Figure 4 (related to Figure 4). Correlation between ddhC and CMPK2 expression in different patient groups in the discovery cohort. 12

Methods S1 – Supplementary items related to STAR Methods metabolite identification (separate file)

Table 1 *Targeted feature extraction related to STAR Methods metabolite identification*

Figure 1 *Tandem mass spectrometry related to STAR Methods metabolite identification*

Figure 2 *Definitive identification of ddhC using a chemical standard related to STAR Methods metabolite identification*

Supplementary Table 1 (related to Figure 1). Patient demographics and sample numbers in discovery and validation cohorts.

DISCOVERY COHORT	Gram +ve bacteraemia	Gram -ve bacteraemia	Pre-COVID-19 viral	COVID-19	Non-infected unwell control	Healthy control	Total
**n (included in at least one assay)**	30	30	29	32	30	13	164
**n (lipid RPC-)**	30	30	29	31	30	13	163
**n (lipid RPC+)**	29	30	29	31	29	12	160
**n (HILIC+)**	29	30	29	31	29	13	161
**Age (median [IQR])**	71 [59-80]	69 [60-79]	41 [26-54]	66 [60-80]	52 [43-68]	34 [31-45]	60 [44-74]
**Female (n [%])**	18 [60]	14 [47]	14 [48]	18 [56]	12 [40]	8 [62]	84 [51]
**WCC x10^9/L (median [IQR])**	11.6 [7.3-14.8]	13.5 [8.9-16.4]	7.8 [5.1-9.5]	7.2 [5.3-9.4]	8.8 [6.6-12.2]	NA	8.9 [6.2-12.9]
**Lymphocyte count x10^9/L (median [IQR])**	0.9 [0.5-1.3]	0.7 [0.5-1.2]	0.6 [0.8-1.0]	1.0 [0.9-1.3]	1.8 [1.0-2.7]	NA	0.9 [0.6-1.5]
**CRP mg/L (median [IQR])**	56 [27-107]	137 [42-205]	31 [17-74]	89 [50-154]	6 [1-15]	NA	56 [15-120]
**VALIDATION COHORT**	**Bacteraemia**		**Viral**		**NA**	**NA**	**Total**
**n (HILIC+)**	40		40		-	-	80
**Age (median [IQR])**	66 [52-77]		55 [45-72]		-	-	61 [48-73]
**Female (n [%])**	23 [58]		20 [50]		-	-	43 [54]

Supplementary Table 1 (related to Figure 1). Patient demographics and sample numbers in discovery and validation cohorts. HILIC = hydrophilic interaction chromatography, RPC = reversed-phase chromatography, IQR = interquartile range, WCC = white cell count, CRP = C-reactive protein, NA = not applicable. In the discovery cohort, routine haematology and biochemistry (WCC, lymphocyte count & CRP) were not available for the 13 healthy controls; CRP values based on 125/151 patients.

Supplementary Table 2 (related to Figure 1). Confirmed pathogens in discovery and validation cohorts.

	DISCOVERY						VALIDATION	
**Patient sub-group**	**Gram +ve bacteraemia**	**Gram -ve bacteraemia**	**Pre-COVID-19 viral**	**COVID-19**	**Non-infected unwell**	**Healthy control**	**Bacterial**	**Viral**
**n (total)**	30	30	29	32	30	13	40	40
Staphylococcus aureus	10	-	-	-	-	-	7	-
Alpha-haemolytic Streptococcus (viridans)	5	-	-	-	-	-	1	-
Streptococcus pneumoniae	4	-	-	-	-	-	2	-
Beta-haemolytic Streptococcus	4	-	-	-	-	-	4	-
Enterococcus faecalis	3	-	-	-	-	-	-	-
Enterococcus casseliflavus	1	-	-	-	-	-	-	-
Listeria monocytogenes	1	-	-	-	-	-	-	-
Clostridium septicum	1	-	-	-	-	-	-	-
Lactobacillus paracasei	1	-	-	-	-	-	-	-
Escherichia coli	-	19	-	-	-	-	21	-
Klebsiella sp.	-	4	-	-	-	-	-	-
Proteus mirabilis	-	2	-	-	-	-	-	-
Enterobacter cloacae	-	2	-	-	-	-	1	-
Serratia marcescens	-	1	-	-	-	-	-	-
Moraxella catarrhalis	-	1	-	-	-	-	-	-
Haemophilus parainfluenzae	-	1	-	-	-	-	-	-
Pseudomonas aeruginosa	-	-	-	-	-	-	2	-
Salmonella typhi	-	-	-	-	-	-	1	-
Acinetobacter junii	-	-	-	-	-	-	1	-
**Influenza A**	-	-	11	-	-	-	-	7
**Adenovirus**	-	-	5	-	-	-	-	1
**Measles**	-	-	3	-	-	-	-	-
**Varicella Zoster Virus**	-	-	3	-	-	-	-	-
**Dengue**	-	-	2	-	-	-	-	-
**Herpes Simplex Virus**	-	-	1	-	-	-	-	3
**Rhinovirus / Adenovirus / Parainf 1***	-	-	1	-	-	-	-	-
**Parainfluenza 2**	-	-	1	-	-	-	-	-
**Herpes Simplex Virus + Adenovirus**	-	-	1	-	-	-	-	-
**Enterovirus**	-	-	1	-	-	-	-	-
**Rhinovirus**	-	-	-	-	-	-	-	6
**Influenza B**	-	-	-	-	-	-	-	5
**Metapneumovirus**	-	-	-	-	-	-	-	2
**Norovirus**	-	-	-	-	-	-	-	1
**Rotavirus**	-	-	-	-	-	-	-	1
**Respiratory Syncytial Virus**	-	-	-	-	-	-	-	1
**Influenza A + Rhinovirus**	-	-	-	-	-	-	-	1
**SARS-CoV-2**	-	-	-	32	-	-		12

Supplementary Table 2 (related to Figure 1). Confirmed pathogens in discovery and validation cohorts. PCR (viral)- or culture (bacterial)-confirmed pathogens in the discovery primary analysis (n=164) and validation (n=80) cohorts. *All three of rhinovirus, parainfluenza 1 virus and adenovirus (weak positive) were detected on a respiratory viral PCR assay for this patient.

Supplementary Table 3 (related to Figure 2). Cross-validation of feature performance using FS-PLS.

	Assay	Most frequent feature	FS-PLS runs (/100)	Median test AUC	IQR test AUC
**Viral vs. other**	Lipid RPC+	778.54/5.01	61	0.843	0.809-0.867
	Lipid RPC-	766.53/5.76	46	0.775	0.724-0.802
	HILIC+	248.06/1.96	100	0.957	0.943-0.970
**Viral vs. bacterial**	Lipid RPC+	711.58/5.90	37	0.833	0.796-0.867
	Lipid RPC-	717.53/6.00	39	0.731	0.701-0.779
	HILIC+	248.06/1.96	99	0.951	0.926-0.969
**Bacterial vs. other**	Lipid RPC+	740.61/6.62	31	0.741	0.710-0.794
	Lipid RPC-	871.69/8.09	30	0.768	0.726-0.813
	HILIC+	512.34/5.09	69	0.797	0.767-0.839

Supplementary Table 3 (related to Figure 2). Cross-validation of feature performance using FS-PLS.
Cross-validation of feature performance in differentiating viral versus other, bacteraemic versus viral and bacteraemic versus other using 100 forward selection-partial least squares (FS-PLS) runs comprising 70:30 training:test splits. FS-PLS runs (/100) = the number of times the feature was selected in 100 FS-PLS runs. ddhC (248.06/1.96) was selected as the discriminating feature in all 100 FS-PLS runs in the HILIC+ assay comparing viral versus other, generating a median test area under the receiver operating characteristic curve (AUC) of 0.957. Features are shown by *m/z*/retention time. IQR = interquartile range, HILIC = hydrophilic interaction chromatography, RPC = reversed-phase chromatography.

Supplementary Table 4 (related to Figure 4). Five gene transcripts most highly correlated with ddhC in the discovery primary analysis cohort.

Gene	Ensembl ID	Correlation with ddhC	p-value
CMPK2	ENSG00000134326	0.763	$< 1x10^{23}$
SPATS2L	ENSG00000196141	0.759	$< 1x10^{23}$
IFI27	ENSG00000165949	0.756	$< 1x10^{23}$
RSAD2 (viperin)	ENSG00000134321	0.748	$< 1x10^{22}$
Unnamed*	ENSG00000233785	0.743	$< 1x10^{21}$

Supplementary Table 4 (related to Figure 4). Five gene transcripts most highly correlated with ddhC in the discovery primary analysis cohort. Pearson's correlation coefficients between log_2-transformed whole blood-derived gene expression and log_2-transformed ddhC intensity, with the five (of 18,248) most highly correlated genes shown (n=122 patients). *No identification available.

Supplementary Figure 1 (related to Figure 2). Discovery cohort metabolomic findings and discovery of ddhC.

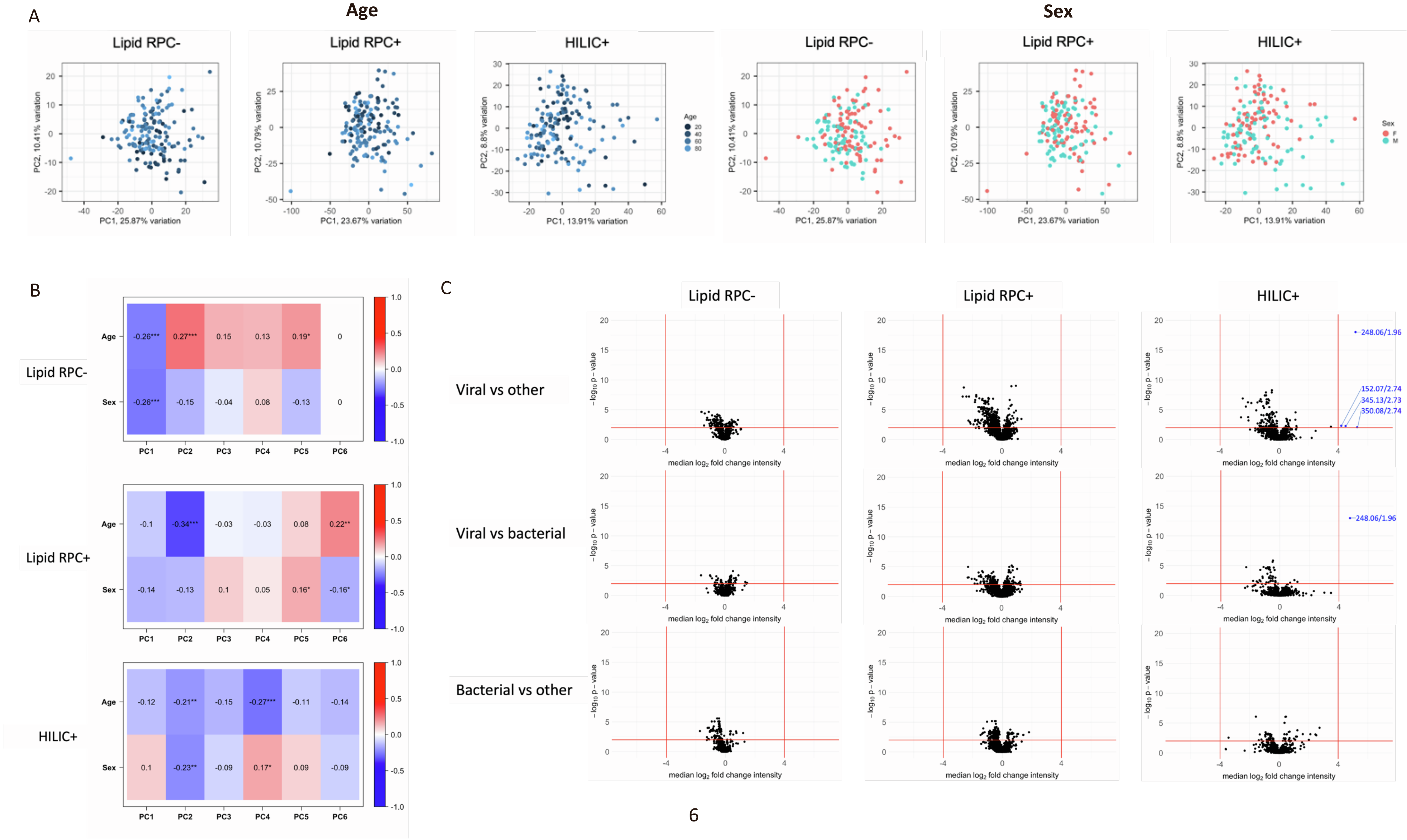

Supplementary Figure 1 (related to Figure 2). Discovery cohort metabolomic findings and discovery of ddhC.

A. Principal component analysis of lipid profiling (lipid RPC-, lipid RPC+) and small molecule profiling (HILIC+) data from discovery primary analysis cohort, showing no variance due to age or sex (n=161-163 samples).

B. Eigencor plots show minimal correlation between principal component variance and age or sex in discovery primary analysis cohort (n=161-163). ***p-value<0.001, **<0.01, *<0.05.

C. Volcano plot showing median $\log_2$ fold change intensity of all features versus $-\log_{10}$ p-value in all assays when comparing: viral versus other, viral versus bacterial, and bacterial versus other groups in discovery primary analysis cohort (n=161-163). Threshold lines in red represent a fold-change of 16 [$\log_2$(foldchange) of 4] and p-value of 0.01 [$-\log_{10}$(p-value) of 2]. Candidate biomarkers are shown in blue by their *m/z*/retention time. P-values generated using the two-sided Wilcoxon test and adjusted using the Benjamini-Hochberg procedure.

HILIC = hydrophilic interaction chromatography, RPC = reversed-phase chromatography.

Supplementary Figure 2 (related to Figure 2). Discovery cohort AUCs and relative feature intensity data for all samples and influence of host demographic and clinical factors on feature intensity.

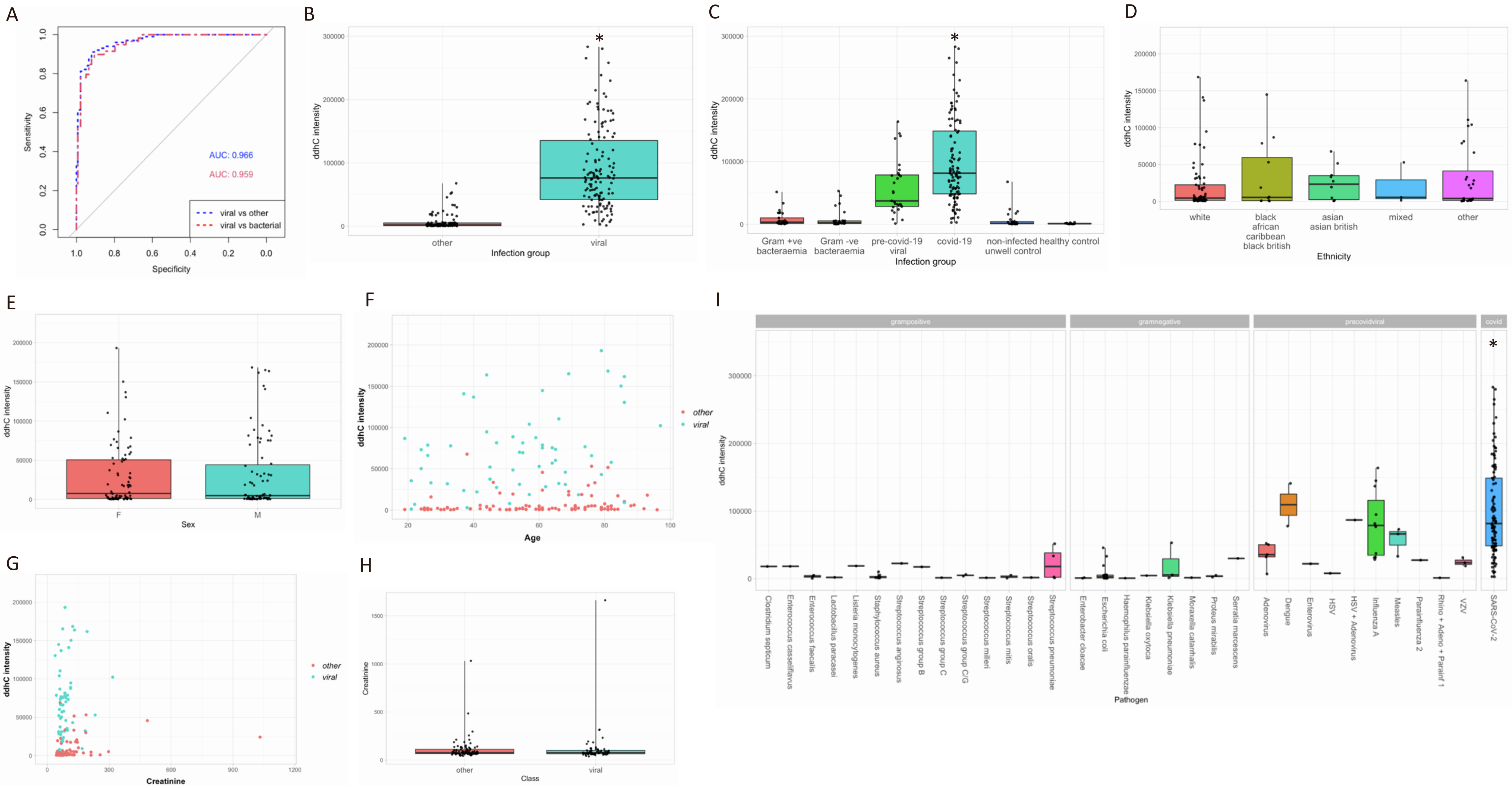

Supplementary Figure 2 (related to Figure 2). Discovery cohort AUCs and relative feature intensity data for all samples and influence of host demographic and clinical factors on feature intensity.
For all plots, ddhC feature *(m/z*/retention time of 248.06/1.96) intensity was used.

A. AUCs for ddhC distinguishing viral (pre-COVID-19 viral and COVID-19) versus all other and viral versus bacterial (Gram-positive and Gram-negative bacteraemia) groups using all discovery cohort samples, including those that spent >5days outside a -80°C freezer. **Blue.** AUC of 0.966 for ddhC differentiating viral infections from all other groups (n=239). **Red.** AUC of 0.959 for ddhC differentiating viral from bacterial infection, with controls omitted (n=197).

B & C. ddhC intensity data in different patient groups using all discovery cohort samples, including those that spent >5days outside a -80°C freezer (n=239). **B.** Viral versus all other groups. **C.** All comparator groups. *4 samples in the COVID-19 group had an intensity of >400000, not shown.

D-H. Effect of ethnicity, sex, age, and renal function on ddhC intensity in the discovery primary analysis cohort. No significant difference in median ddhC intensity between **D.** ethnicity groups (n=123 with available ethnicity data) or **E.** sex (n=161), p-value threshold <0.01. **F.** No significant correlation between ddhC intensity and age in the discovery primary analysis cohort (n=161). **G.** Low correlation between ddhC intensity and serum creatinine (n=148, healthy controls not included as creatinine not available, Pearson correlation coefficient 0.555, p-value $<1x10^{-12}$). **H.** No significant difference in median creatinine between viral and other groups (n=148), p-value threshold <0.01. 2 samples in the COVID-19 group had a relative intensity of >700000, not shown in any D-H plots. P-values for median comparisons generated using the two-sided Wilcoxon test (E, H) and Kruskal-Wallis test (D).

I. Effect of causative pathogen on serum ddhC intensity in the total discovery cohort. Points represent individual patients (including samples that spent >5days outside a -80°C freezer, n=239). *4 samples in the COVID-19 group had an intensity of >400000, not shown.

Scatter plots: non-viral group (red points) includes bacteraemic patients, non-infected unwell controls, and healthy controls; viral group (blue points) includes COVID-19 and pre-COVID-19 viral infection patients. Box plots: points represent individual patients; boxes represent interquartile ranges with medians.

Supplementary Figure 3 (related to Figure 3). Validation cohort metabolomic findings and severity analysis.

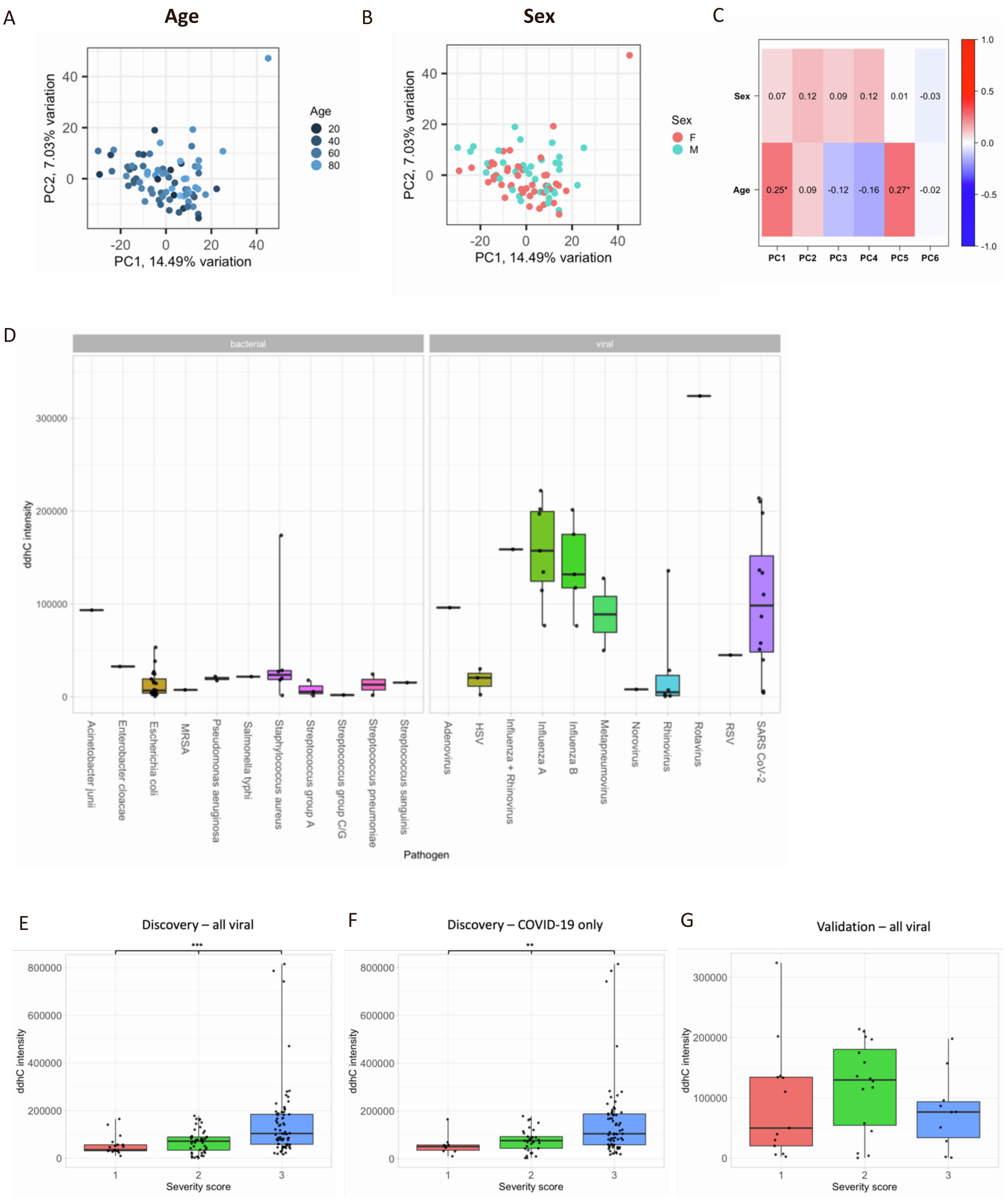

Supplementary Figure 3 (related to Figure 3). Validation cohort metabolomic findings and severity analysis.

A & B. Principal component analysis of small molecule profiling (HILIC+) data from validation cohort, showing no variance due to age or sex (n=80).

C. Eigencor plot shows minimal correlation between principal component variance and age or sex in validation cohort (n=80). *p-value<0.05.

D. ddhC intensity by pathogen type in validation cohort (n=80).

E-G. Severity analysis in discovery and validation cohorts. Association between median ddhC intensity (y axis) and outcome severity in all viral cases (pre-COVID-19 viral and COVID-19) in the total discovery patient cohort (**E**) (n=138), ***p-value $<1x10^{-5}$, and in COVID-19 cases only (**F**) (n=109), in the total discovery patient cohort **p-value $<1x10^{-3}$. **G.** No significant association found between outcome severity and ddhC intensity in all viral cases in the validation cohort (n=40). Severity score: 1 = admission duration 0-2 days, 2 = admission duration 3-8 days, 3 = admission duration >8 days, ICU admission or death. p-values generated using the Kruskal-Wallis test.

Box plots: points represent individual patients; boxes represent interquartile ranges with medians.

Supplementary Figure 4 (related to Figure 4). Correlation between ddhC and *CMPK2* expression in different patient groups in the discovery cohort.

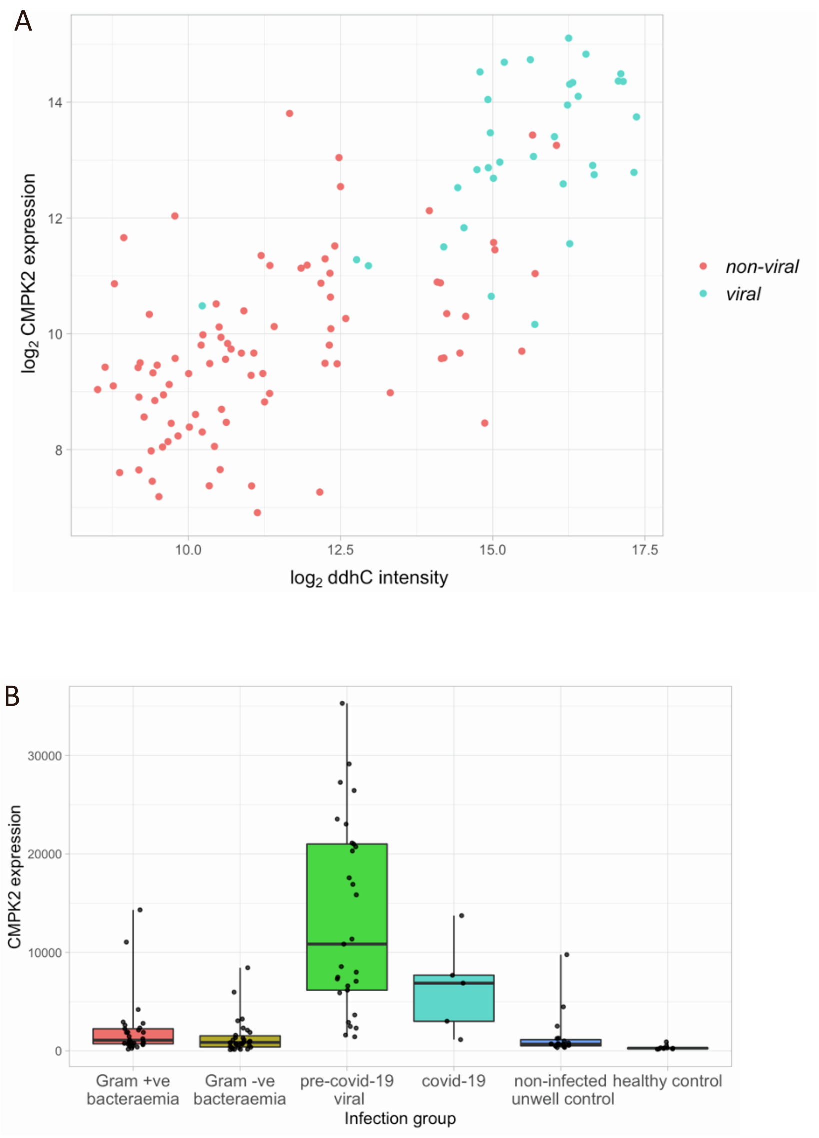

Supplementary Figure 4 (related to Figure 4). Correlation between ddhC intensity and CMPK2 expression in different patient groups in the discovery cohort.

A. Correlation between ddhC intensity and *CMPK2* gene expression in 122 patients in the discovery cohort. Non-viral group (red points) includes bacteraemic patients, non-infected unwell controls, and healthy controls; viral group (blue points) includes COVID-19 and pre-COVID-19 viral infection patients. Pearson correlation coefficient = 0.763, p-value < $1x10^{-23}$.

B. Normalised *CMPK2* gene counts for 122 patients in different infection groups. Points represent individual patients. Boxes represent interquartile ranges with medians.
