# Supplementary material for: Antiviral metabolite 3′-deoxy-3′,4′-didehydro-cytidine is detectable in serum and identifies acute viral infections including COVID-19
Source: Med. 2022 Mar 11;3(3):204–215.e6. doi: 10.1016/j.medj.2022.01.009 (PMC8801973; doi:10.1016/j.medj.2022.01.009)
Supplement: Methods S1. Supplementary items related to STAR Methods metabolite identification — Table 1. Targeted feature extraction related to STAR Methods metabolite identification. Figure 1. Tandem MS related to STAR Methods metabolite identification. Figure 2. Definitive identification of ddhC using a chemical standard related to STAR Methods metabolite identification. [file mmc2.zip › Methods S1/Methods S1 Figure 2.docx]

## **Supplementary Methods Figure 2 (related to STAR Methods metabolite identification). Definitive identification of ddhC using a chemical standard.**

A


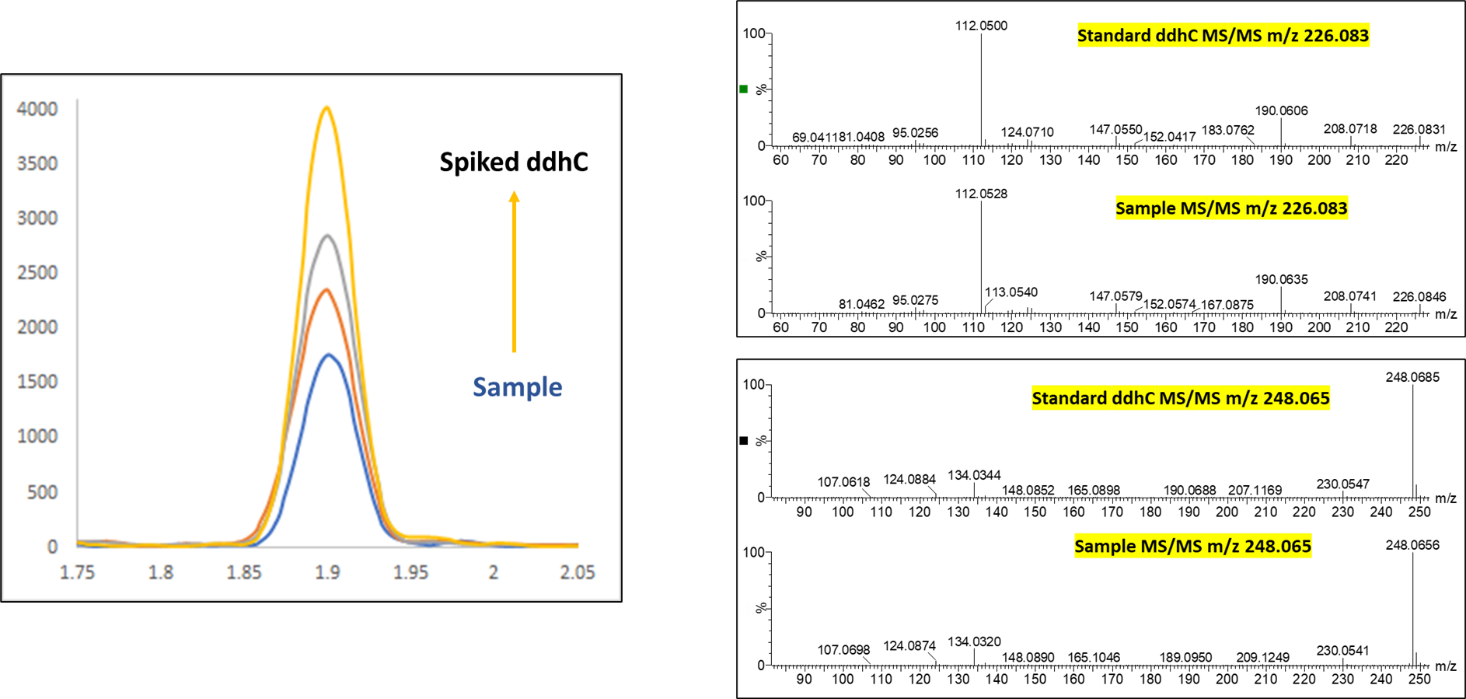

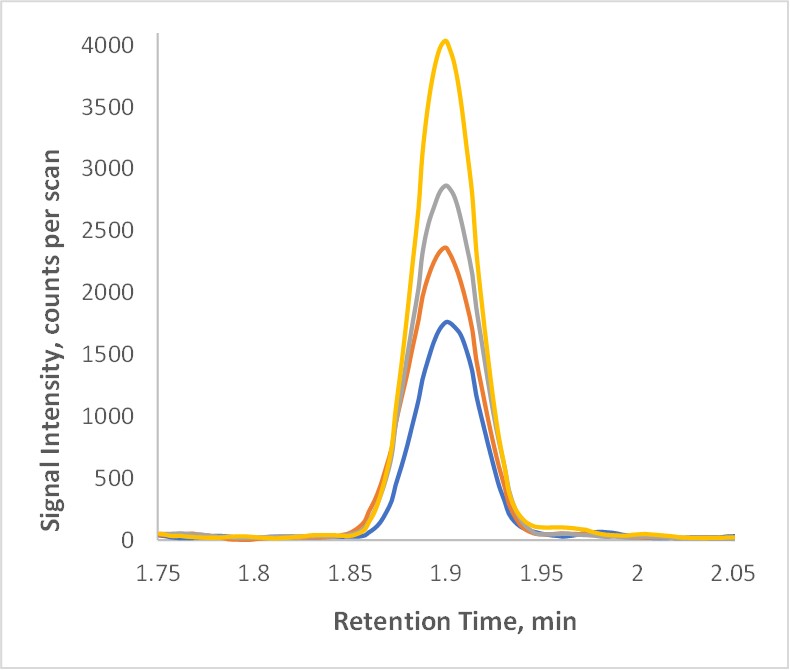


C

B

**Supplementary Methods Figure 2 (related to STAR Methods metabolite identification). Definitive identification of ddhC using a chemical standard.** **A.** Hydrophilic interaction chromatography (HILIC)-derived chromatographic retention time of the feature of interest in the pooled sample (blue) matches that of the pooled serum sample spiked with different concentrations of ddhC chemical standard (red = 2.5ng/mL, grey = 5ng/mL, yellow = 10ng/mL). **B.** MS/MS spectra of sample [M+H]^+^ ion and **C**. MS/MS spectra of sample [M+Na]^+^ ion match the fragmentation patterns of the ddhC standard.
