# Supplementary material for: Antiviral metabolite 3′-deoxy-3′,4′-didehydro-cytidine is detectable in serum and identifies acute viral infections including COVID-19
Source: Med. 2022 Mar 11;3(3):204–215.e6. doi: 10.1016/j.medj.2022.01.009 (PMC8801973; doi:10.1016/j.medj.2022.01.009)
Supplement: Methods S1. Supplementary items related to STAR Methods metabolite identification — Table 1. Targeted feature extraction related to STAR Methods metabolite identification. Figure 1. Tandem MS related to STAR Methods metabolite identification. Figure 2. Definitive identification of ddhC using a chemical standard related to STAR Methods metabolite identification. [file mmc2.zip › Methods S1/Methods S1 Table 1.docx]

## **Supplementary Methods Table 1 (related to STAR Methods metabolite identification). Targeted feature extraction.**

| Ion type | *m/z* | AUC viral vs all other groups |
| --- | --- | --- |
| First isotope [M+Na]^+^ | 249.0676 | 0.943 |
| [M+H]^+^ | 226.0827 | 0.948 |
| [M+K]^+^ | 264.0383 | 0.951 |
| In-source fragment | 112.0517 | 0.953 |

###

**Supplementary Methods Table 1 (related to STAR Methods metabolite identification).** **Targeted feature extraction.** Area under curve (AUC) comparing serum from viral infections versus all other groups in the hydrophilic interaction chromatography (HILIC+) data set for the primary analysis cohort (n = 161) for all features representing the same metabolite as the identified feature of interest (248.06/1.96).
