# Supplementary material for: Antiviral metabolite 3′-deoxy-3′,4′-didehydro-cytidine is detectable in serum and identifies acute viral infections including COVID-19
Source: Med. 2022 Mar 11;3(3):204–215.e6. doi: 10.1016/j.medj.2022.01.009 (PMC8801973; doi:10.1016/j.medj.2022.01.009)
Supplement: Methods S1. Supplementary items related to STAR Methods metabolite identification — Table 1. Targeted feature extraction related to STAR Methods metabolite identification. Figure 1. Tandem MS related to STAR Methods metabolite identification. Figure 2. Definitive identification of ddhC using a chemical standard related to STAR Methods metabolite identification. [file mmc2.zip › Methods S1/Methods S1 Figure 1.docx]

## **Supplementary Methods Figure 1 (related to STAR Methods metabolite identification). Tandem mass spectrometry.**


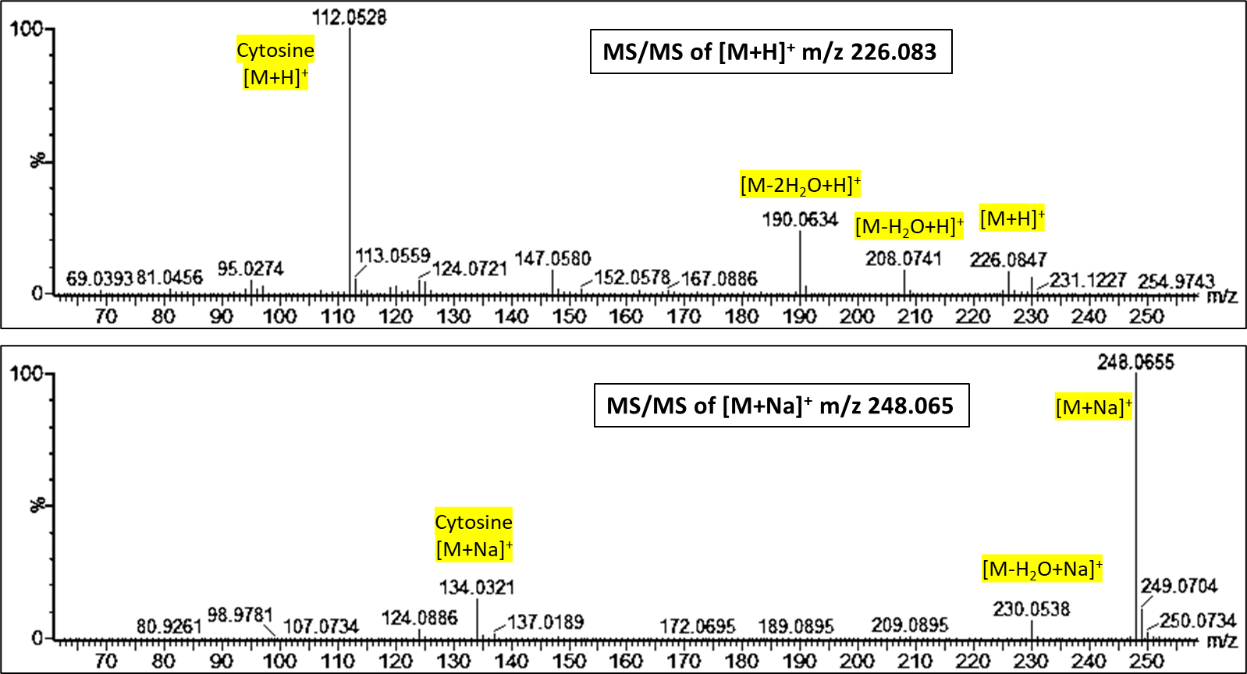


**Supplementary Methods Figure 1 (related to STAR Methods metabolite identification).** **Tandem mass spectrometry.** MS/MS spectrum obtained with collision energy ramp of 10-45V for [M+H]^+^ ion m/z 226.0827 (upper panel) and [M+Na]^+^ ion m/z 248.0647 (lower panel) using a study sample with high intensity of these features.
