# Supplementary material for: Antiviral metabolite 3′-deoxy-3′,4′-didehydro-cytidine is detectable in serum and identifies acute viral infections including COVID-19
Source: Med. 2022 Mar 11;3(3):204–215.e6. doi: 10.1016/j.medj.2022.01.009 (PMC8801973; doi:10.1016/j.medj.2022.01.009)
Supplement: Document S2. Article plus supplemental information [file mmc3.pdf]

Med

CellPress OPEN ACCESS

Clinical and Translational Report

Antiviral metabolite 3′-deoxy-3′,4′-didehydro-cytidine is detectable in serum and identifies acute viral infections including COVID-19

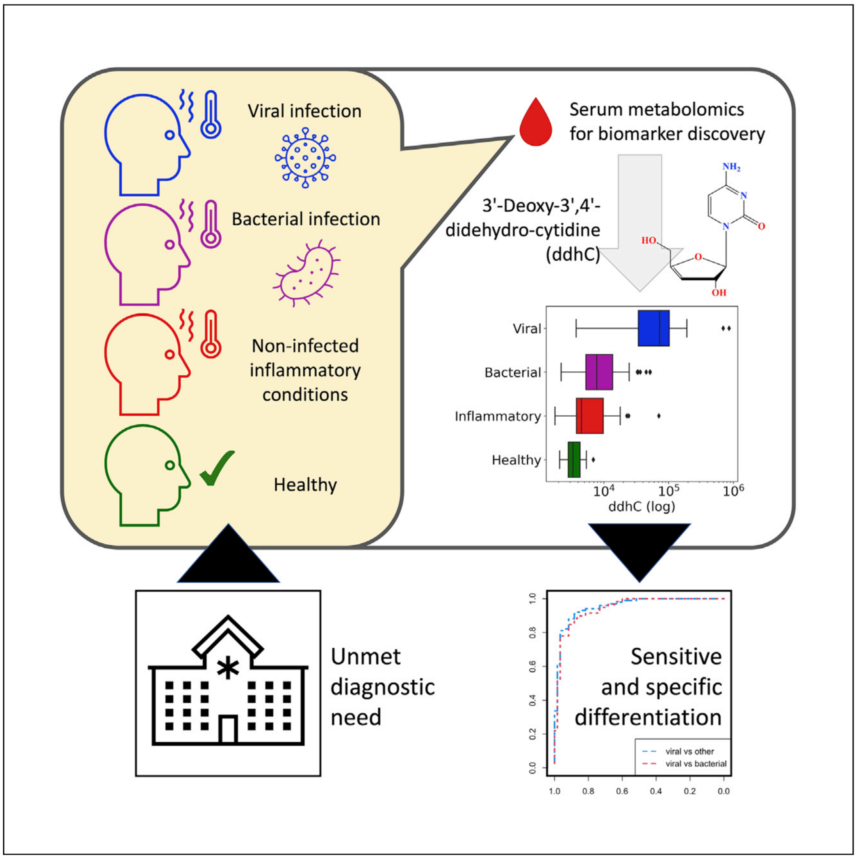

Mehta et al. perform untargeted metabolic profiling of serum samples from patients presenting to the emergency department with different infections. The antiviral small molecule 3′-deoxy-3′,4′-didehydro-cytidine (ddhC) was detectable in human serum and accurately differentiated viral infections from other infection syndromes in both a discovery and validation patient cohort.

Translation to Patients

Ravi Mehta, Elena Chekmeneva, Heather Jackson, ..., Matthew R. Lewis, Zoltan Takats, Shiranee Sriskandan

s.sriskandan@imperial.ac.uk

Highlights

Global metabolic profiling of serum from patients with suspected infection presenting to ED

Single metabolite differentiates viral from bacterial infections and other unwell controls

Identified as ddhC, a free base of only known human antiviral small molecule

ddhC has a key potential role as rapid diagnostic for viral infections

Mehta et al., Med 3, 204–215
March 11, 2022 © 2022 The Author(s). Published by Elsevier Inc.
https://doi.org/10.1016/j.medj.2022.01.009

CellPress OPEN ACCESS

Med

Clinical and Translational Report

Antiviral metabolite 3′-deoxy-3′,4′-didehydro-cytidine is detectable in serum and identifies acute viral infections including COVID-19

Ravi Mehta,[1] Elena Chekmeneva,[2,3,8] Heather Jackson,[1,8] Caroline Sands,[2,3] Ewurabena Mills,[1] Dominique Arancon,[4] Ho Kwong Li,[1,5] Paul Arkell,[1,4] Timothy M. Rawson,[1,4,6] Robert Hammond,[4] Maisarah Amran,[4] Anna Haber,[4] Graham S. Cooke,[1] Mahdad Noursadeghi,[6] Myrsini Kaforou,[1] Matthew R. Lewis,[2,3] Zoltan Takats,[2,3] and Shiranee Sriskandan[1,5,7,9,*]

SUMMARY

Background: There is a critical need for rapid viral infection diagnostics to enable prompt case identification in pandemic settings and support targeted antimicrobial prescribing.

Methods: Using untargeted high-resolution liquid chromatography coupled with mass spectrometry, we compared the admission serum metabolome of emergency department patients with viral infections (including COVID-19), bacterial infections, inflammatory conditions, and healthy controls. Sera from an independent cohort of emergency department patients admitted with viral or bacterial infections underwent profiling to validate findings. Associations between whole-blood gene expression and the identified metabolite of interest were examined.

Findings: 3′-Deoxy-3′,4′-didehydro-cytidine (ddhC), a free base of the only known human antiviral small molecule ddhC-triphosphate (ddhCTP), was detected for the first time in serum. When comparing 60 viral with 101 non-viral cases in the discovery cohort, ddhC was the most significantly differentially abundant metabolite, generating an area under the receiver operating characteristic curve (AUC) of 0.954 (95% CI: 0.923–0.986). In the validation cohort, ddhC was again the most significantly differentially abundant metabolite when comparing 40 viral with 40 bacterial cases, generating an AUC of 0.81 (95% CI 0.708–0.915). Transcripts of viperin and *CMPK2*, enzymes responsible for ddhCTP synthesis, were among the five genes most highly correlated with ddhC abundance.

Conclusions: The antiviral precursor molecule ddhC is detectable in serum and an accurate marker for acute viral infection. Interferon-inducible genes viperin and *CMPK2* are implicated in ddhC production *in vivo*. These findings highlight a future diagnostic role for ddhC in viral diagnosis, pandemic preparedness, and acute infection management.

Funding: NIHR Imperial BRC; UKRI.

Context and Significance

Researchers at Imperial College London measured the small molecules in serum samples from a cohort of adult patients admitted to hospital with different types of infection, including COVID-19, other viruses, and bacteria. They found that the molecule "ddhC" was more abundant in patients with viral infections than in those without. This result was replicated in a separate validation patient cohort. ddhC was previously shown to have antiviral properties in laboratory cells but has not to date been detected in living humans. Using ddhC to rapidly diagnose viral infections could prove beneficial in early recognition and containment of future pandemics and help to reduce unnecessary antibiotic use.

INTRODUCTION

Early differentiation of acute infectious etiologies is now a priority in diagnostic innovation. Conventional methods relying on pathogen identification through culture, PCR, or antigen detection are time-consuming and/or insensitive, leading to diagnostic delays that result in inappropriate antimicrobial prescription and infection

 © 2022 The Author(s). Published by Elsevier Inc.
This is an open access article under the CC BY license (http://creativecommons.org/licenses/by/4.0/).

transmission.[1–3] There is therefore renewed interest in novel biomarkers of infection classes that can better guide therapeutic and infection control decisions in real time.

Technologies for large-scale characterization of low-molecular-weight metabolites have the potential to aid discovery of novel biomarkers of infectious diseases. Liquid chromatography coupled with mass spectrometry (LC-MS) and nuclear magnetic resonance spectroscopy stand out among the most commonly employed techniques in the field. The use of MS has already revolutionized modern microbiology by enabling rapid detection of bacterial species from cultured colonies.[4]

Despite its growing impact on biomedical research, metabolic profiling of biofluids has produced candidate biomarkers in only a small number of infectious states. One study identified a two-metabolite serum signature differentiating infected from non-infected patients within a systemic inflammatory response syndrome cohort.[5] Metabolomic interrogation of cerebrospinal fluid from patients with meningitis was able to differentiate between *Mycobacterium tuberculosis* and other infectious causes.[6] Wang et al. examined the lipidome of 40 patients in a pediatric cohort prior to the COVID-19 pandemic and identified a 3-lipid signature that discriminated bacterial from viral infection, although wider metabolomic changes were not reported.[7] A number of more recent studies report metabolic differences between patients with and without SARS-CoV-2 infection,[8–10] but comparator groups did not include bacterial infections.

We investigated the serum metabolome of adult patients presenting to two UK emergency departments with a range of suspected infection syndromes, including COVID-19, to derive and cross-validate novel biomarkers for viral and bacterial infections. We used point-of-admission samples to replicate the time point where a discovered biomarker would be used clinically. To ensure diagnostic certainty, we adopted a case-control approach with laboratory-proven infections. Our sampling included unwell, non-infected cases to ensure that any biomarkers identified accounted for cases of inflammatory conditions unrelated to infection.[11]

RESULTS

Samples used for discovery and validation cohorts

In the discovery cohort, serum from 232 patients and 13 healthy controls underwent LC-MS-based metabolic profiling (Figure 1), for the measurement of small molecules and lipids using hydrophilic interaction liquid chromatography (HILIC) and reversed-phase chromatography (RPC), respectively. All discovery cohort patients were admitted via one of two emergency departments at Imperial College Healthcare NHS Trust with a suspected acute infection syndrome. A total of 173 patients were from the Imperial arm of the Bioresource for Adult Infectious Diseases (BioAID) study (30 Gram-positive bacteremia, 30 Gram-negative bacteremia, 30 pre-COVID-19 viral, 53 COVID-19, 30 non-infected unwell controls), and 59 patients were from the Imperial Microbial Products in Infection study (all COVID-19). Owing to insufficient sample volume or data quality, four samples were excluded from the discovery primary analysis in both lipid profiling assays, and five from the small molecule profiling assay. Eighty of the 112 COVID-19 samples were not transferred to a −80 °C freezer within 5 days of collection and so this sub-group was also excluded from the discovery primary analysis. In all assays, principal component analysis (PCA) did not show clustering of samples by age or sex and eigencor plots did not show correlation above 0.4 (Figures S1A and S1B). The final number of samples included

[1]Department of Infectious Disease, Imperial College London, London W12 0NN, UK
[2]National Phenome Centre, Imperial College London, London SW7 2AZ, UK
[3]Division of Systems Medicine, Department of Metabolism, Digestion and Reproduction, Imperial College London, London SW7 2AZ, UK
[4]Imperial College Healthcare NHS Trust, London W12 0HS, UK
[5]MRC Centre for Molecular Bacteriology & Infection, Imperial College London, London SW7 2AZ, UK
[6]Division of Infection & Immunity, University College London, London WC1 E6BT, UK
[7]NIHR Health Protection Research Unit in Healthcare-associated Infection & Antimicrobial Resistance, Imperial College London, London W12 0NN, UK
[8]These authors contributed equally
[9]Lead contact
*Correspondence: s.sriskandan@imperial.ac.uk
https://doi.org/10.1016/j.medj.2022.01.009

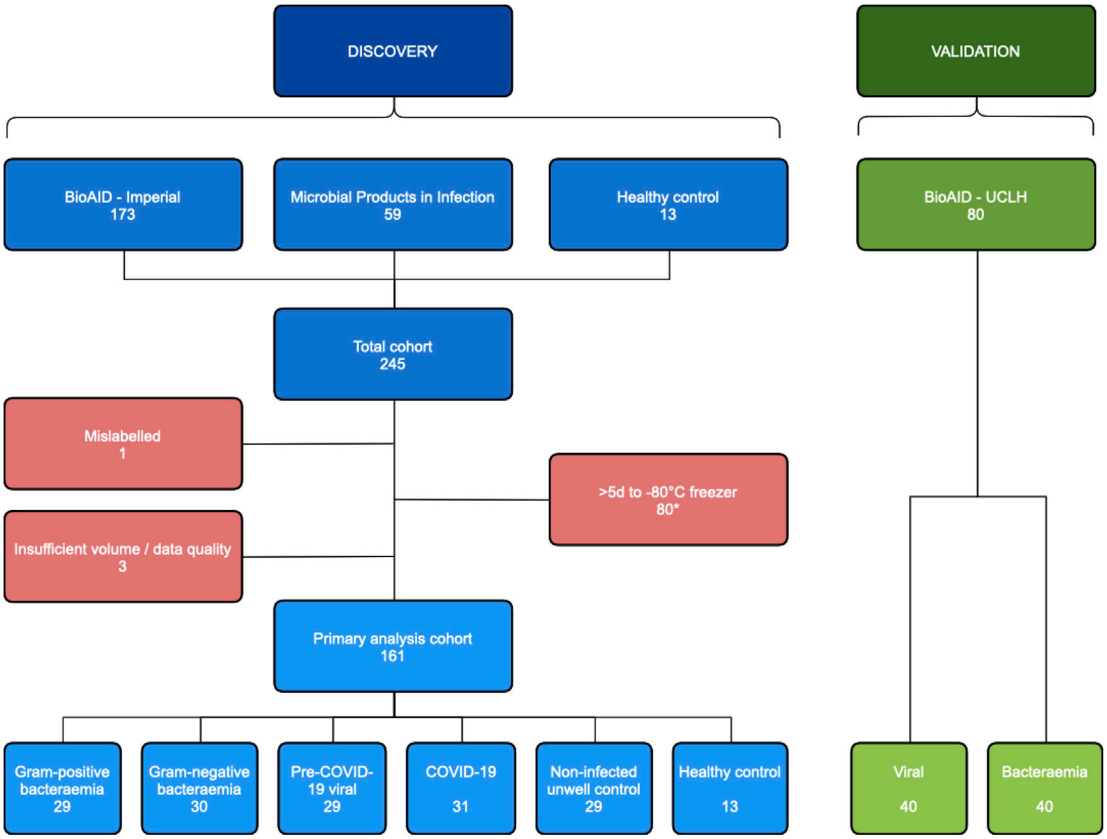

Figure 1. Study flowchart

Flowchart of sample selection and exclusion for the discovery primary analysis and validation cohorts; data shown for the small molecule profiling (HILIC+) assay. Eighty discovery COVID-19 samples were excluded as they were transferred to a −80 °C freezer >5 days after collection (*two samples excluded within this group also had insufficient volume/data quality).

in the discovery primary analysis ranged from 161 to 163, depending on the assay. In a separate validation cohort, 80 patients were selected from the University College London Hospital (UCLH) arm of the BioAID study (40 viral, 40 bacteremia); no samples were excluded. Patient demographic data and confirmed infection pathogens for the discovery and validation cohorts are shown in Tables S1 and S2, respectively.

Metabolic profiling of discovery cohort identifies ddhC as best-performing discriminator for viral infections

Analysis of the discovery cohort small molecule profiling dataset identified several significantly differentially abundant (SDA) features with a median absolute $\log_2$ fold-change of >4 and p value <0.01 when comparing viral cases (pre-COVID-19 viral and COVID-19) versus all other groups, and viral versus bacterial cases (Gram-positive and Gram-negative bacteremia) (Figures 2A and 2B). The top SDA discriminator was the feature 248.0647 *m/z* at 1.96 min, which showed a 36-fold change in the median intensity in viral cases compared with all other groups (adjusted p value $<1 \times 10^{-18}$). This metabolite was identified as 3′-deoxy-3′,4′-didehydro-cytidine (ddhC), a free base of the ribonucleotide ddhC-triphosphate (ddhCTP) recently reported to have antiviral properties *in vitro*.[12] Its identity was confirmed by comparison to a chemical reference standard using LC with tandem MS (LC-MS/MS; Methods Figures S1 and S2). Using the same empirical thresholds, no SDA features were identified in bacterial cases versus all other groups in the small

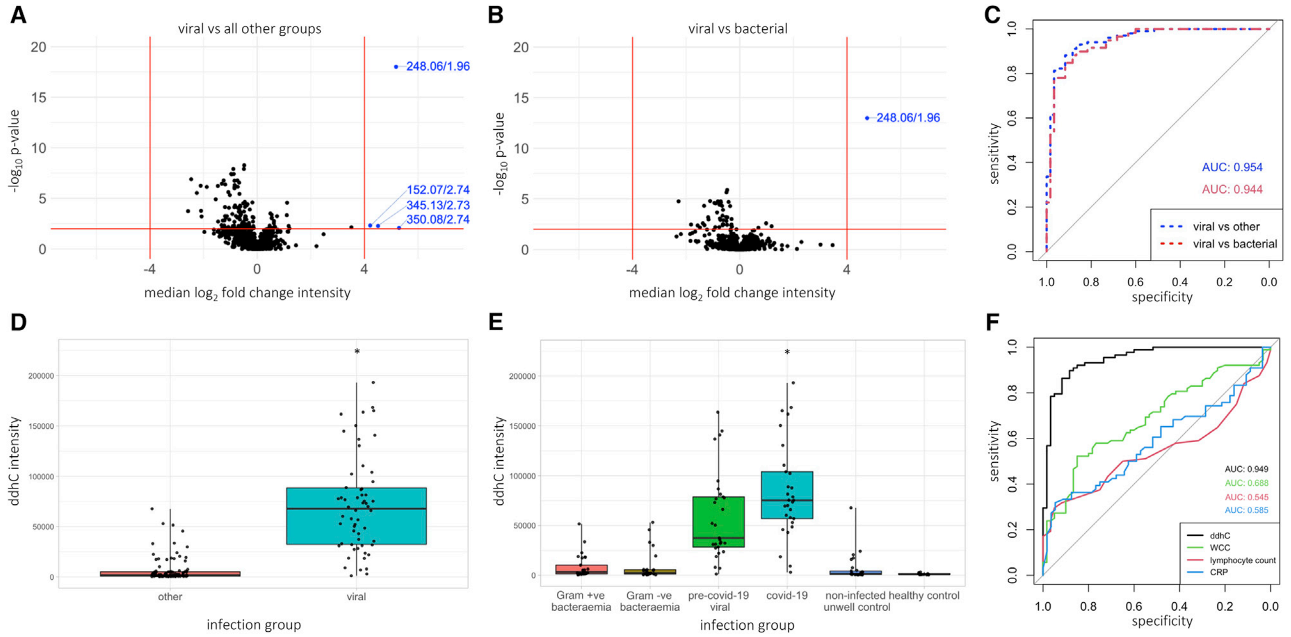

Figure 2. ddhC as the best-performing discriminator for viral infections in the discovery cohort

(A and B) Volcano plots showing median log_2 fold change in intensity of each feature versus -log_{10} *p* value in the discovery primary analysis cohort small molecule profiling dataset (n = 161) when comparing (A) viral cases (pre-COVID-19 viral and COVID-19) versus all other groups, and (B) viral versus bacterial (Gram-positive and Gram-negative bacteremia) cases, with controls omitted. Empirical threshold lines in red represent a fold-change of 16 (log_2[fold-change] of 4) and *p* value of 0.01 ($-log_{10}$[*p* value] of 2). Candidate biomarkers are shown in blue by mass:charge ratio/retention time, with 248.06/1.96 (ddhC) performing best. *p*-values generated using the two-sided Wilcoxon test and adjusted using the Benjamini-Hochberg procedure.
(C) AUCs for ddhC distinguishing viral versus all other and viral versus bacterial groups in the discovery primary analysis cohort. Blue. AUC of 0.954 (95% CI 0.923-0.986) for ddhC differentiating viral infections from all other groups (n = 161). Red. AUC of 0.944 (95% CI 0.905-0.983) for ddhC differentiating viral from bacterial infections, with controls omitted (n = 119).
(D and E) Relative ddhC intensity data in different patient groups in the discovery primary analysis cohort (n = 161). Points represent individual patients. Boxes represent IQRs with medians. (D) Viral versus all other groups. (E) Individual comparator groups. *2 samples in the COVID-19 group had a relative intensity of >700,000, not shown.
(F) Comparison of AUCs between ddhC, white cell count (WCC), lymphocyte count, and CRP as biomarkers to distinguish viral infections from all other groups in the discovery primary analysis cohort. Black - ddhC (AUC = 0.949 [95% CI 0.914-0.983], n = 148); green - WCC (AUC = 0.688 [95% CI 0.603-0.774], n = 148); red - lymphocyte count (AUC = 0.545 [95% CI 0.452-0.637], n = 148); blue - CRP (AUC = 0.585 [95 CI 0.483-0.687], n = 122). Healthy controls not included, as WCC, lymphocyte count, and CRP not available.

molecule profiling dataset, nor were any identified in either of the lipid profiling datasets (Figure S1C).

Using all samples from the discovery primary analysis cohort, ddhC returned an area under the receiver operating characteristic curve (AUC) of 0.954 (95% CI 0.923-0.986; sensitivity 88.1%, specificity 91.7%) in discriminating viral infections from all other groups, and 0.944 (95% CI 0.905-0.983; sensitivity 89.8%, specificity 86.7%) in discriminating viral from bacterial infections (Figure 2C). When we included the sub-group of samples that spent more than 5 days outside a −80 °C freezer, similar results were achieved with AUCs of 0.966 and 0.959, respectively (Figure S2A).

In the discovery primary analysis cohort, ddhC demonstrated a higher relative median intensity among patients with viral infections compared with other groups (Figures 2D and 2E). Similar results were achieved when including samples that spent more than 5 days outside a −80 °C freezer (Figures S2B and S2C). There was no correlation between ddhC and age, and no significant difference in the median ddhC intensity between sex and ethnicity subgroups (Figures S2D, S2E, and S2F). There

was a low correlation between admission serum creatinine and ddhC (correlation coefficient 0.555, *p* value $<1 \times 10^{-12}$), but there was no significant difference in the median serum creatinine between viral and non-viral groups (Figures S2G and S2H). ddhC intensities associated with specific pathogens are presented in Figure S2I.

Cross-validation using single-feature forward selection-partial least squares

To assess and cross-validate the discriminatory performance of single markers distinguishing infection groups in the data, we used the forward selection-partial least squares (FS-PLS) method. For each comparison (viral versus other, viral versus bacterial, bacterial versus other) we present the discriminating feature that was selected most frequently out of 100 different training:test FS-PLS runs and the median and interquartile range (IQR) of the test AUCs generated (Table S3). When comparing viral versus all other groups, ddhC (small molecule feature 248.06/1.96) was selected in all 100 FS-PLS runs, and generated a median (IQR) test AUC of 0.957 (0.943-0.970). When comparing viral versus bacterial groups, ddhC (small molecule feature 248.06/1.96) was selected in 99 of 100 FS-PLS runs, generating a median (IQR) test AUC of 0.951 (0.926-0.971).

ddhC performs better than white cell count, lymphocyte count, and C-reactive protein as a biomarker for viral infections

We compared the ability of ddhC to differentiate viral infection from other groups to alternative biomarkers, such as white cell count, lymphocyte count, and C-reactive protein (CRP), which were taken as part of routine admission clinical laboratory tests. We used the small molecule profiling data from the discovery primary analysis cohort (n = 161) and excluded healthy controls (n = 13), for whom there were no routine laboratory test data (n = 148 in total). All patients had a white cell count and lymphocyte count recorded, and 122 of 148 patients had a CRP. Routine admission clinical laboratory tests performed poorly compared with ddhC (Figure 2F).

ddhC differentiates viral versus bacterial infections in an independent validation cohort

Sera from a separate cohort of 80 patients from the UCLH arm of the BioAID study with confirmed viral (n = 40) and bacterial infections (n = 40) underwent untargeted small molecule profiling. PCA did not show clustering of samples by age or sex and the corresponding eigencor plots did not show correlation above 0.3 (Figures S3A, S3B and S3C). Using the same empirical thresholds as the discovery analysis, no SDA discriminators were found. Adjusting the median absolute $\log_2$ fold-change threshold to 2, the top SDA discriminator was the feature 248.0648 *m/z* at 1.93 min, which is the same ion $[M + Na]^+$ of ddhC identified in the discovery cohort and showed a 6.7-fold change in the median intensity in the viral compared with bacterial group (Figure 3A, adjusted *p* value $<1 \times 10^{-3}$). ddhC returned an AUC of 0.811 (95% CI 0.708-0.915, sensitivity 72.5%, specificity 92.5%) in discriminating viral from bacterial infections (Figure 3B) and demonstrated a higher relative intensity among patients with viral compared with bacterial infections (Figure 3C). ddhC intensities associated with specific pathogens are presented in Figure S3D.

ddhC intensity associates with outcome severity in viral infections in the discovery but not validation cohort

To assess the role of ddhC as a prognostic indicator, we performed a post hoc exploratory analysis of the ddhC response in all viral infections (pre-COVID-19 viral and COVID-19) categorized by outcome severity. We used the total discovery patient cohort (including samples that were transferred to −80 °C storage after 5 days, n = 138) to maximize power to differentiate between categories. The median

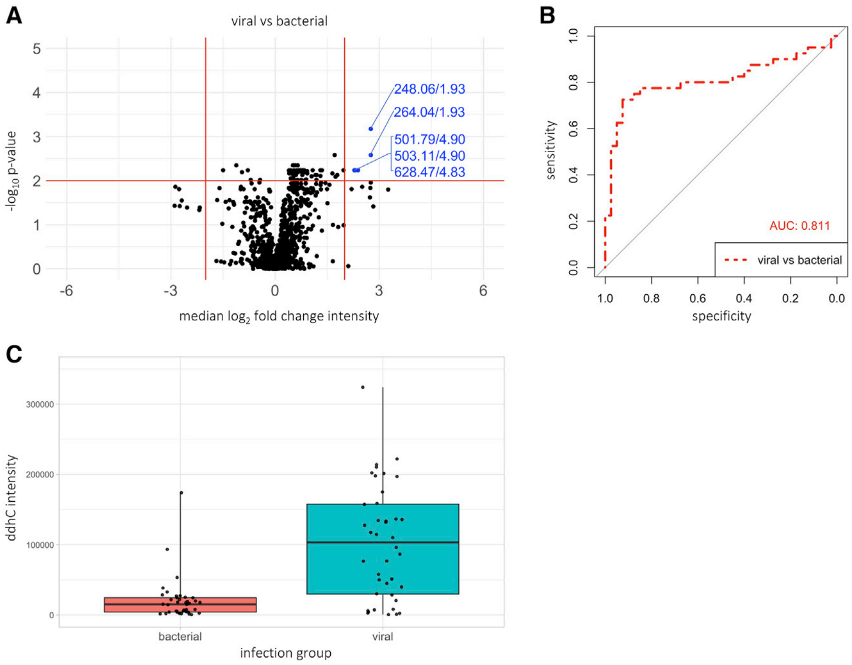

Figure 3. ddhC differentiates viral versus bacterial infections in an independent validation cohort
Data from the validation cohort of sera from 40 viral and 40 bacterial infection patients undergoing the small molecule profiling assay. (A) volcano plot showing median $\log_2$ fold change in intensity of each feature versus -$\log_{10}$ *p* value when comparing viral versus bacterial patients. Empirical threshold lines in red represent a fold-change of 4 [$\log_2$(fold-change) of 2] and *p* value of 0.01 [-$\log_{10}$(*p* value) of 2]. Features exceeding the threshold are shown in blue by mass:charge ratio/retention time, with 248.06/1.92 (ddhC) performing best. The next best performing feature 264.04/1.93 also corresponds to ddhC ($[M+K]^+$ adduct). *p*-values generated using the two-sided Wilcoxon test and adjusted using the Benjamini-Hochberg procedure.
(B) Area under the receiver operating characteristic curve of 0.811 (95% CI 0.708-0.915) for ddhC discriminating between viral and bacterial infections.
(C) Relative ddhC intensity data in viral versus bacterial groups. Points represent individual patients. Boxes represent interquartile ranges with medians

relative ddhC intensity was 35,525 in mild disease (admission duration 0–2 days), increasing to 71,569 in moderate (admission duration 3–8 days) and 103,995 in severe disease (admission duration >8 days, intensive care unit [ICU] admission or death); *p* value $<1 \times 10^{-5}$ (Figure S3E). A similar trend was seen in COVID-19 patients alone (Figure S3F). This association was, however, not replicated in the validation cohort, where no significant difference was found between severity groups in 40 patients with viral infection (Figure S3G).

ddhC intensity is associated with gene expression of viperin and *CMPK2* in whole blood

RNA-sequencing (RNA-seq) data were available from 122 patients in the discovery small molecule profiling primary analysis cohort (29 Gram-positive bacteremia, 30 Gram-negative bacteremia, 29 pre-COVID-19-viral, five COVID-19, 19 non-infected unwell controls, 10 healthy controls). The correlation between $\log_2$-transformed ddhC intensity and counts for 18,248 genes was evaluated. The five gene transcripts with the greatest correlation to ddhC intensity are listed in Table S4. Two of the five genes are directly implicated in ddhCTP metabolism–*RSAD2* (viperin), aided by *CMPK2*, mediates ddhCTP production during viral infection.[12] The correlation coefficient for viperin expression and ddhC intensity was 0.748 (*p* value $<1 \times 10^{-22}$);

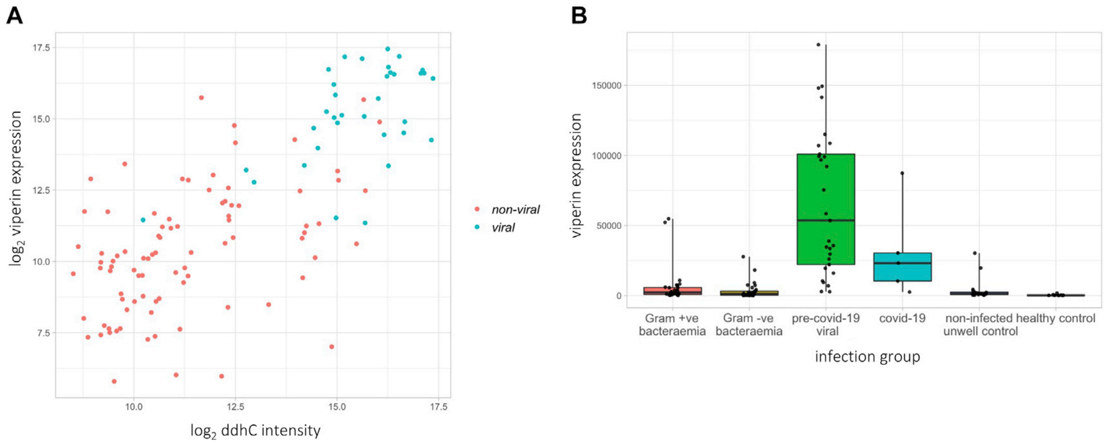

Figure 4. ddhC intensity is associated with gene expression of viperin in whole blood

(A) Correlation between ddhC intensity and viperin (*RSAD2*) gene expression in 122 patients in the discovery cohort. Non-viral group (red points) includes bacteremic patients, non-infected unwell controls, and healthy controls; viral group (blue points) includes COVID-19 and pre-COVID-19 viral infection patients. Pearson correlation coefficient = 0.748, p value $<1 \times 10^{-22}$.

(B) Normalized viperin gene counts for 122 patients in different infection groups. Points represent individual patients. Boxes represent IQRs with medians.

viperin was more highly expressed in patients with viral infections (Figure 4). Data for *CMPK2* showed the same trends (Figure S4).

DISCUSSION

The ability to rapidly differentiate infection syndromes is an urgent requirement, underlined by the ongoing COVID-19 pandemic and the growing threat of antimicrobial resistance. We capitalized on the sensitivity of high-resolution LC coupled with MS to discover that ddhC, a free base of the antiviral molecule ddhCTP, was detectable in patient serum. In a discovery cohort, ddhC was found to have a 36-fold higher median intensity in patients with viral infections, including COVID-19, compared with those with bacterial infections, non-infected inflammatory states, and healthy controls, corresponding to an AUC of 0.954, sensitivity of 88.1% and specificity of 91.7%. It outperformed white cell count, lymphocyte count, and CRP as a viral biomarker (AUCs of 0.688, 0.545, and 0.585, respectively). In an independent validation cohort, ddhC was again the most significantly differentially abundant metabolite when comparing patients with viral versus bacterial infections, generating an AUC of 0.811.

ddhCTP has recently been shown to be the first and, to the best of our knowledge, only small molecule produced by humans that is capable of directly inhibiting viral replication machinery.[12] Gizzi et al. showed that the enzyme viperin (virus inhibitory protein, endoplasmic reticulum-associated, interferon-inducible), aided by the genomically adjacent enzyme cytidylate monophosphate kinase 2 (*CMPK2*), catalyzes the conversion of CTP to ddhCTP, which acts as a chain terminator for multiple viral RNA-dependent RNA polymerases (RdRPs).[12] Synthetic ddhC traversed the plasma membrane of Vero and HEK293T cells, suggesting a mechanism for how ddhC might eventually reach the serum in detectable quantity. ddhC has also been detected in prokaryotic cells; *Escherichia coli* production of ddhCTP after viperin homolog expression was associated with T7 phage RdRP suppression, suggesting a role for ddhCTP in bacterial immunity to viruses.[13] To our knowledge, ddhC has hitherto not been identified in human or other mammalian serum, nor associated with COVID-19. We showed that this antiviral molecule was a sensitive and specific serum biomarker for a range of viral infections, including COVID-19, in clinical samples of

CellPress
OPEN ACCESS

patients presenting to hospital. In a subset of patients for whom RNA-seq data were available, we showed that viperin and *CMPK2* expression was also increased in patients with viral infections. Furthermore, of more than 18,000 genes, their expression was among the top five most highly correlated with ddhC intensity (correlation coefficients 0.75 and 0.76, respectively), providing a plausible mechanism by which ddhC may be produced during viral infection.

A robust serum biomarker of viral infection would provide real-time determination of infectious etiology, aiding patient triage and decision-making regarding antimicrobial prescription. It could prove vital in infection prevention and control measures, especially in the context of a viral pandemic, where rapid detection of an acute viral illness, not dependent on nucleotide amplification via PCR, would enable prompt patient isolation while awaiting definitive pathogen identification. In our study, we excluded patients who had definite co-infection, i.e. pathogens detected in more than one infection category. However, it is possible that some patients were not tested and thus had undetected viral and bacterial co-infections, which may explain why a small proportion of our patients with bacterial infections demonstrated a moderate ddhC response. Using ddhC in conjunction with a serum bacterial biomarker, such as procalcitonin, may be a powerful strategy to help rapidly detect a co-infection and mitigate the risk of not treating a bacterial infection.

ddhC was again the most significantly differentially abundant small metabolite when comparing viral and bacterial cases in the validation cohort, although did not perform as well as in the discovery cohort, demonstrating a lower sensitivity for viral infections. The reasons underlying this difference are unclear. It is possible that certain viruses may not evoke or may actively limit the ddhC response. For example, all four cases of HSV infection showed a low ddhC intensity: three were in the validation and one in the discovery cohort, which may have contributed to the observed difference in AUCs. Second, if as expected, the concentration of ddhC rises and falls like other acute phase reactants, differences in the timing of sample collection post symptom onset may also have affected its accuracy. Future work comparing larger cohorts of patients with specific viral infections, with sampling at different time points post infection onset, will help investigate these hypotheses. Our unadjusted, exploratory analysis of severity in the discovery cohort showed that the level of ddhC at the point of hospital admission was associated with increased length of stay, requirement for ICU support, and death, suggesting a potential prognostic role for ddhC. This result was not replicated in the validation cohort. Whether a prognostic role for ddhC is reserved for certain viral infections such as COVID-19, which was over-represented in the discovery compared with the validation cohort, awaits further investigation.

The antiviral properties of ddhCTP rely on inhibition of viral RdRPs and are demonstrable *in vitro*, raising the possibility that it may have therapeutic action. RdRPs are an enticing target for novel antivirals and are an active focus of ongoing antiviral therapeutics research,[14] as there are no functional homologs in uninfected human cells, thus off-target drug effects are less likely. Wood et al. demonstrated that ddhCTP can be robustly synthesized on a gram scale, facilitating further investigation of its use.[15] Seifert et al. showed that exogenous ddhCTP can inhibit SARS-CoV-2 polymerase activity in Huh7-hACE2 cells, although it did not decrease SARS-CoV-2 N-protein immunofluorescence.[16] Future work will ascertain whether exogenous and/or endogenous ddhCTP can inhibit SARS-CoV-2 viral replication in other cell lines, and its effect on other viruses. Here we show that ddhC is produced naturally *in vivo* in response to viral infections, at levels that are detectable

in the circulation, increasing the likelihood of an acceptable safety profile of ddhCTP as a therapeutic.

Our study demonstrates a number of strengths. We deliberately included both healthy and unhealthy non-infected controls, reducing the likelihood of selecting biomarkers confounded by inflammation unrelated to infection. We used stringent inclusion criteria for infected patients, excluding those in whom the timing of clinical presentation or PCR/culture result might have affected infection status at the point of sample acquisition. We used admission-day samples taken prior to any intervention, the time point where a diagnostic test would be most useful.

In conclusion, using high-fidelity metabolic profiling of serum from patients attending hospital, we found that the antiviral molecule ddhC is present in human serum during viral infection and represents an accurate biomarker for a wide range of viral infections, including COVID-19. These findings pave the way for a universal blood test to rapidly identify acute viral infections, which could play a key role in both pandemic preparedness and routine acute infection management.

Limitations of study

Our study should be viewed in the context of its limitations. First, to ensure diagnostic certainty, we only included bacterial infections associated with bacteremia, and did not include fungal and protozoal infections. We plan to assess the performance of ddhC in new patient cohorts that include a wider range of infection syndromes. Second, we allowed a period of up to 5 days from serum sample acquisition to −80 °C storage for the discovery cohort, which may have introduced variability in potential metabolite degradation at 4 °C during the 0- to 5-day period. However, ddhC performed similarly in COVID-19 samples that had not been frozen within 5 days, which suggests it is likely to be a robust marker that is not highly susceptible to temperature-associated degradation and suitable for "real-life" biochemical analytics, where samples may require time to reach laboratories prior to testing. The serum samples for both arms of the discovery cohort were collected in the same way from the clinical diagnostic laboratory, albeit that patients were prospectively recruited to BioAID, while retrospectively recruited to Microbial Products in Infection. Third, our severity analysis was exploratory without consideration of potential confounders and incorporated hospitalization duration, which can be affected by factors other than severity. Fourth, we did not have access to the patients' pre-admission medication history, which could potentially affect the serum metabolic profile, but we do not suspect this would significantly differ between viral and non-viral groups. Fifth, our cohort represents patients unwell enough to seek hospital attention, further work will be required to assess the role of ddhC in less unwell patients presenting to primary care and determine whether it is detectable in minimally invasive samples such as urine or saliva.

STAR★METHODS

Detailed methods are provided in the online version of this paper and include the following:

- KEY RESOURCES TABLE
- RESOURCE AVAILABILITY
  - Lead contact
  - Materials availability
  - Data code and availability

CellPress
OPEN ACCESS

- EXPERIMENTAL MODEL AND SUBJECT DETAILS
  - Study design and population
  - Discovery cohort patient selection
  - Validation cohort patient selection
- METHOD DETAILS
  - Metabolic profiling assays
  - Sample preparation and data pre-processing for metabolic profiling
  - Metabolite identification
  - Multi-omic comparison
- QUANTIFICATION AND STATISTICAL ANALYSIS

SUPPLEMENTAL INFORMATION

Supplemental information can be found online at https://doi.org/10.1016/j.medj.2022.01.009.

ACKNOWLEDGMENTS

The authors acknowledge the NHS and NIHR CRN clinical research teams, diagnostic laboratory leads, and data managers whose work supports BioAID at Imperial College Healthcare NHS Trust and University College Hospital NHS Trust. The authors also acknowledge Ash Salam, Stephane Camuzeaux, Benjamin Cooper, and Lynn Maslen, National Phenome Center, for their technical and administrative expertise in running the LC-MS experiments, and Lachlan Coin for the development of the FS-PLS algorithm.

R.M. and T.M.R. are NIHR Academic Clinical Fellows and, with S.S. and G.S.C., acknowledge support from the NIHR Imperial BRC, which also supports the Leonard and Dora Colebrook laboratory used for bioresourcing; M.K. is a Sir Henry Wellcome Fellow (206508/Z/17/Z); H.J. receives support from the Wellcome Trust (4-Year PhD program, grant number 215214/Z/19/Z); H.K.L. is a Medical Research Council (MRC) CMBI Clinical Research Training Fellow (MR/R502376/1); T.M.R. acknowledges the Center for Antimicrobial Optimisation, Imperial College London; G.S.C. is an NIHR Research Professor; M.N. is a Wellcome Trust Investigator (207511/Z/17/Z) and acknowledges the NIHR UCLH BRC; S.S. acknowledges the NIHR Health Protection Research Unit (HPRU) in Healthcare-associated Infections & AMR; M.R.L., Z.T., C.S., and E.C. receive support from the MRC (grant number MC_PC_12025) and metabolomics infrastructure support was provided by the NIHR Imperial BRC. Human control samples used in this research project were obtained from a subcollection of the Imperial College Healthcare Tissue Bank (ICHTB), which is supported by the NIHR Imperial BRC. ICHTB is approved by Wales REC3 to release human material for research (17/WA/0161).

Funding: NIHR Imperial Biomedical Research Centre; MRC (UKRI).

AUTHOR CONTRIBUTIONS

S.S., M.N., and G.S.C. established the BioAID cohort. R.M., S.S., M.R.L., M.N., and Z.T. conceived and designed the study. R.M., H.K.L., and E.M. selected and prepared samples from the discovery cohort for analysis. R.M. and M.N. selected and prepared samples from the validation cohort for analysis. R.M., M.N., P.A., T.M.R., R.H., M.A., A.H., and D.A. acquired clinical data. C.S. facilitated LC-MS pre-processing. R.M., C.S., and H.J. verified the underlying data. R.M., H.J., M.K., and C.S. had unrestricted access to and analyzed the data. E.C. facilitated metabolite identification. R.M. wrote the first draft of the manuscript. S.S., M.K., H.J., M.R.L., E.C., M.N., G.C., and C.S. edited the manuscript. All authors agreed to submit the manuscript,

read and approved the final draft, and take full responsibility of its content, including the accuracy of the data.

DECLARATION OF INTERESTS

The authors declare no competing interests.

Received: July 28, 2021
Revised: November 14, 2021
Accepted: January 21, 2022
Published: January 31, 2022

REFERENCES

1. Vandenberg, O., Martiny, D., Rochas, O., van Belkum, A., and Kozlakidis, Z. (2021). Considerations for diagnostic COVID-19 tests. Nat. Rev. Microbiol. *19*, 171–183. https://doi.org/10.1038/s41579-020-00461-z.

2. Kanji, J.N., Zelyas, N., MacDonald, C., Pabbaraju, K., Khan, M.N., Prasad, A., Hu, J., Diggle, M., Berenger, B.M., and Tipples, G. (2021). False negative rate of COVID-19 PCR testing: a discordant testing analysis. Virol. J. *18*, 13. https://doi.org/10.1186/s12985-021-01489-0.

3. Denny, K.J., Gartside, J.G., Alcorn, K., Cross, J.W., Maloney, S., and Keijzers, G. (2019). Appropriateness of antibiotic prescribing in the emergency department. J. Antimicrob. Chemother. *74*, 515–520. https://doi.org/10.1093/jac/dky447.

4. Singhal, N., Kumar, M., Kanaujia, P.K., and Virdi, J.S. (2015). MALDI-TOF mass spectrometry: an emerging technology for microbial identification and diagnosis. Front. Microbiol. *6*, 791. https://doi.org/10.3389/fmicb.2015.00791.

5. Neugebauer, S., Giamarellos-Bourboulis, E.J., Pelekanou, A., Marioli, A., Baziaka, F., Tsangaris, I., Bauer, M., and Kiehntopf, M. (2016). Metabolite profiles in sepsis: developing prognostic tools based on the type of infection. Crit. Care Med. *44*, 1649–1662. https://doi.org/10.1097/CCM.0000000000001740.

6. Zhang, P., Zhang, W., Lang, Y., Qu, Y., Chen, J., and Cui, L. (2019). (1)H nuclear magnetic resonance-based metabolic profiling of cerebrospinal fluid to identify metabolic features and markers for tuberculosis meningitis. Infect. Genet. Evol. *68*, 253–264. https://doi.org/10.1016/j.meegid.2019.01.003.

7. Wang, X., Nijman, R., Camuzeaux, S., Sands, C., Jackson, H., Kaforou, M., Emonts, M., Herberg, J.A., Maconochie, I., Carrol, E.D., et al. (2019). Plasma lipid profiles discriminate bacterial from viral infection in febrile children. Sci. Rep. *9*, 17714. https://doi.org/10.1038/s41598-019-53721-1.

8. Overmyer, K.A., Shishkova, E., Miller, I.J., Balnis, J., Bernstein, M.N., Peters-Clarke, T.M., Meyer, J.G., Quan, Q., Muehlbauer, L.K., Trujillo, E.A., et al. (2021). Large-scale multi-omic analysis of COVID-19 severity. Cell Syst. *12*, 23–40.e27. https://doi.org/10.1016/j.cels.2020.10.003.

9. Shen, B., Yi, X., Sun, Y., Bi, X., Du, J., Zhang, C., Quan, S., Zhang, F., Sun, R., Qian, L., et al. (2020). Proteomic and metabolomic characterization of COVID-19 patient sera. Cell *182*, 59–72.e15. https://doi.org/10.1016/j.cell.2020.05.032.

10. Kimhofer, T., Lodge, S., Whiley, L., Gray, N., Loo, R.L., Lawler, N.G., Nitschke, P., Bong, S.H., Morrison, D.L., Begum, S., et al. (2020). Integrative modeling of quantitative plasma lipoprotein, metabolic, and amino acid data reveals a multiorgan pathological signature of SARS-CoV-2 infection. J. Proteome Res. *19*, 4442–4454. https://doi.org/10.1021/acs.jproteome.0c00519.

11. Lindahl, A., Forshed, J., and Nordstrom, A. (2016). Overlap in serum metabolic profiles between non-related diseases: implications for LC-MS metabolomics biomarker discovery. Biochem. Biophys. Res. Commun. *478*, 1472–1477. https://doi.org/10.1016/j.bbrc.2016.08.155.

12. Gizzi, A.S., Grove, T.L., Arnold, J.J., Jose, J., Jangra, R.K., Garforth, S.J., Du, Q., Cahill, S.M., Dulyaninova, N.G., Love, J.D., et al. (2018). A naturally occurring antiviral ribonucleotide encoded by the human genome. Nature *558*, 610–614. https://doi.org/10.1038/s41586-018-0238-4.

13. Bernheim, A., Millman, A., Ofir, G., Meitav, G., Avraham, C., Shomar, H., Rosenberg, M.M., Tal, N., Melamed, S., Amitai, G., and Sorek, R. (2021). Prokaryotic viperins produce diverse antiviral molecules. Nature *589*, 120–124. https://doi.org/10.1038/s41586-020-2762-2.

14. Zhu, W., Chen, C.Z., Gorshkov, K., Xu, M., Lo, D.C., and Zheng, W. (2020). RNA-dependent RNA polymerase as a target for COVID-19 drug discovery. SLAS Discov. *25*, 1141–1151. https://doi.org/10.1177/2472555220942123.

15. Wood, J.M., Evans, G.B., Grove, T.L., Almo, S.C., Cameron, S.A., Furneaux, R.H., and Harris, L.D. (2021). Chemical synthesis of the antiviral nucleotide analogue ddhCTP. J. Org. Chem. *86*, 8843–8850. https://doi.org/10.1021/acs.joc.1c00761.

16. Seifert, M., Bera, S.C., van Nies, P., Kirchdoerfer, R.N., Shannon, A., Le, T.-T.-N., Meng, X., Xia, H., Wood, J.M., Harris, L.D., et al. (2021). Inhibition of SARS-CoV-2 polymerase by nucleotide analogs from a single-molecule perspective. Elife *10*, e70968. https://doi.org/10.7554/eLife.70968.

17. Coin, L.J. (2018). Lachlancoin/fspls: Minimal TB Biomarkers (Version 0.5.1) (Zenodo).

18. Blighe, K. Lun, A. (2020). PCAtools: Everything Principal Components Analysis. https://github.com/kevinblighe/PCAtools

19. Robin, X., Turck, N., Hainard, A., Tiberti, N., Lisacek, F., Sanchez, J.C., and Muller, M. (2011). pROC: an open-source package for R and S+ to analyze and compare ROC curves. BMC Bioinformatics *12*, 77. https://doi.org/10.1186/1471-2105-12-77.

20. Chambers, M.C., Maclean, B., Burke, R., Amodei, D., Ruderman, D.L., Neumann, S., Gatto, L., Fischer, B., Pratt, B., Egertson, J., et al. (2012). A cross-platform toolkit for mass spectrometry and proteomics. Nat. Biotechnol. *30*, 918–920. https://doi.org/10.1038/nbt.2377.

21. Smith, C.A., Want, E.J., O'Maille, G., Abagyan, R., and Siuzdak, G. (2006). XCMS: processing mass spectrometry data for metabolite profiling using nonlinear peak alignment, matching, and identification. Anal. Chem. *78*, 779–787. https://doi.org/10.1021/ac051437y.

22. Sands, C.J., Wolfer, A.M., Correia, G.D.S., Sadawi, N., Ahmed, A., Jimenez, B., Lewis, M.R., Glen, R.C., Nicholson, J.K., and Pearce, J.T.M. (2019). The nPYc-Toolbox, a Python module for the pre-processing, quality-control and analysis of metabolic profiling datasets. Bioinformatics *35*, 5359–5360. https://doi.org/10.1093/bioinformatics/btz566.

23. Wolfer, A.M., Correia, G.D.S., Sands, C.J., Camuzeaux, S., Yuen, A.H.Y., Chekmeneva, E., Takats, Z., Pearce, J.T.M., and Lewis, M.R. (2021). peakPantheR, an R package for large-scale targeted extraction and integration of annotated metabolic features in LC-MS profiling datasets. Bioinformatics. https://doi.org/10.1093/bioinformatics/btab433.

24. Shallcross, L.J., Mentzer, A., Rahman, S., Cooke, G.S., Sriskandan, S., and Noursadeghi, M. (2018). Cohort study protocol: Bioresource in adult infectious diseases (BioAID). Wellcome Open Res. *3*, 97. https://doi.org/10.12688/wellcomeopenres.14690.1.

25. Li, H.K., Kaforou, M., Rodriuez-Manzano, J., Channon-Wells, S., Moniri, A., Habgood-Coote, D., Gupta, R.K., Mills, E.A., Arancon, D., Lin, J., et al. (2021). Discovery and validation of a 3-gene transcriptional signature to distinguish COVID-19 and other viral infections from bacterial sepsis in adults; a case-control then observational cohort study. Lancet

CellPress
OPEN ACCESS

Microbe. https://doi.org/10.2139/ssrn.3766286.

26. Lewis, M.R., Pearce, J.T., Spagou, K., Green, M., Dona, A.C., Yuen, A.H., David, M., Berry, D.J., Chappell, K., Horneffer-van der Sluis, V., et al. (2016). Development and application of ultra-performance liquid chromatography-TOF MS for precision large scale urinary metabolic phenotyping. Anal. Chem. *88*, 9004–9013. https://doi.org/10.1021/acs.analchem.6b01481.

27. Izzi-Engbeaya, C., Comninos, A.N., Clarke, S.A., Jomard, A., Yang, L., Jones, S., Abbara, A., Narayanaswamy, S., Eng, P.C., Papadopoulou, D., et al. (2018). The effects of kisspeptin on beta-cell function, serum metabolites and appetite in humans. Diabetes Obes. Metab. *20*, 2800–2810. https://doi.org/10.1111/dom.13460.

28. Sands, C.J., Gomez-Romero, M., Correia, G., Chekmeneva, E., Camuzeaux, S., Izzi-Engbeaya, C., Dhillo, W.S., Takats, Z., and Lewis, M.R. (2021). Representing the metabolome with high fidelity: range and response as quality control factors in LC-MS-based global profiling. Anal. Chem. *93*, 1924–1933. https://doi.org/10.1021/acs.analchem.0c03848.

29. Wishart, D.S., Feunang, Y.D., Marcu, A., Guo, A.C., Liang, K., Vazquez-Fresno, R., Sajed, T., Johnson, D., Li, C., Karu, N., et al. (2018). Hmdb 4.0: the human metabolome database for 2018. Nucleic Acids Res. *46*, D608–D617. https://doi.org/10.1093/nar/gkx1089.

30. Guijas, C., Montenegro-Burke, J.R., Domingo-Almenara, X., Palermo, A., Warth, B., Hermann, G., Koellensperger, G., Huan, T., Uritboonthai, W., Aisporna, A.E., et al. (2018). METLIN: a technology platform for identifying knowns and unknowns. Anal. Chem. *90*, 3156–3164. https://doi.org/10.1021/acs.analchem.7b04424.

31. Mass Spectrometry Data Center. NIST/EPA/NIH Mass Spectral Library. https://chemdata.nist.gov/dokuwiki/doku.php?id=chemdata:nist17.

32. Lamble, S., Batty, E., Attar, M., Buck, D., Bowden, R., Lunter, G., Crook, D., El-Fahmawi, B., and Piazza, P. (2013). Improved workflows for high throughput library preparation using the transposome-based Nextera system. BMC Biotechnol. *13*, 104. https://doi.org/10.1186/1472-6750-13-104.

33. Andrews, S. (2010). FastQC: A Quality Control Tool for High Throughput Sequence Data, https://www.bioinformatics.babraham.ac.uk/projects/fastqc/.

34. Ewels, P., Magnusson, M., Lundin, S., and Kaller, M. (2016). MultiQC: summarize analysis results for multiple tools and samples in a single report. Bioinformatics *32*, 3047–3048. https://doi.org/10.1093/bioinformatics/btw354.

35. Quinlan, A.R., and Hall, I.M. (2010). BEDTools: a flexible suite of utilities for comparing genomic features. Bioinformatics *26*, 841–842. https://doi.org/10.1093/bioinformatics/btq033.

36. Dobin, A., Davis, C.A., Schlesinger, F., Drenkow, J., Zaleski, C., Jha, S., Batut, P., Chaisson, M., and Gingeras, T.R. (2013). STAR: ultrafast universal RNA-seq aligner. Bioinformatics *29*, 15–21. https://doi.org/10.1093/bioinformatics/bts635.

37. Li, H., Handsaker, B., Wysoker, A., Fennell, T., Ruan, J., Homer, N., Marth, G., Abecasis, G., and Durbin, R.; Genome Project Data Processing, Subgroup (2009). The sequence alignment/map format and SAMtools. Bioinformatics *25*, 2078–2079. https://doi.org/10.1093/bioinformatics/btp352.

38. Liao, Y., Smyth, G.K., and Shi, W. (2014). featureCounts: an efficient general purpose program for assigning sequence reads to genomic features. Bioinformatics *30*, 923–930. https://doi.org/10.1093/bioinformatics/btt656.

39. Howe, K.L., Achuthan, P., Allen, J., Allen, J., Alvarez-Jarreta, J., Amode, M.R., Armean, I.M., Azov, A.G., Bennett, R., Bhai, J., et al. (2021). Ensembl 2021. Nucleic Acids Res. *49*, D884–D891. https://doi.org/10.1093/nar/gkaa942.

40. Zhang, Y., Parmigiani, G., and Johnson, W.E. (2020). ComBat-seq: batch effect adjustment for RNA-seq count data. NAR Genom Bioinform *2*. https://doi.org/10.1093/nargab/lqaa078.

41. Love, M.I., Huber, W., and Anders, S. (2014). Moderated estimation of fold change and dispersion for RNA-seq data with DESeq2. Genome Biol. *15*, 550. https://doi.org/10.1186/s13059-014-0550-8.

42. R Core Team (2020). R: A Language and Environment for Statistical Computing (R Foundation for Statistical Computing).

43. Benjamini, Y., and Hochberg, Y. (1995). Controlling the false discovery rate: a practical and powerful approach to multiple testing. J. R. Stat. Soc. Ser. B (Methodol.) *57*, 289–300. https://doi.org/10.1111/j.2517-6161.1995.tb02031.x.

44. Gliddon, H.D., Kaforou, M., Alikian, M., Habgood-Coote, D., Zhou, C., Oni, T., Anderson, S.T., Brent, A.J., Crampin, A.C., Eley, B., et al. (2021). Identification of reduced host transcriptomic signatures for tuberculosis disease and digital PCR-based validation and quantification. Front. Immunol. *12*, 637164. https://doi.org/10.3389/fimmu.2021.637164.

45. Youden, W.J. (1950). Index for rating diagnostic tests. Cancer *3*, 32–35. https://doi.org/10.1002/1097-0142(1950)3:1<32::AID-CNCR2820030106>3.0.CO;2-3.

STAR★METHODS

KEY RESOURCES TABLE

REAGENT or RESOURCE	SOURCE	IDENTIFIER
**Biological samples**		
Patient sera	Bioresource for Adult Infectious Diseases (BioAID) and Microbial Products	N/A
**Chemicals, peptides, and recombinant proteins**		
3′-Deoxy-3′,4′-didehydro-cytidine (ddhC) chemical standard	Berry & Associates	PY7790
**Deposited data**		
Metabolomic data	MetaboLights	[MetaboLights]:[MTBLS718]
RNASeq data used for correlation	MetaboLights	[MetaboLights]:[MTBLS718]
**Software and algorithms**		
Forward selection-partial least squares analysis	Coin[17]	Zenodo Lachlancoin/fspls: Minimal TB Biomarkers (Version 0.5.1)
PCATools	Blighe and Lun[18]	https://github.com/kevinblighe/PCAtools
pROC	Robin et al.[19]	https://www.ncbi.nlm.nih.gov/pubmed/21414208
ProteoWizard	Chambers et al.[20]	https://www.ncbi.nlm.nih.gov/pubmed/23051804
XCMS	Smith et al.[21]	https://www.ncbi.nlm.nih.gov/pubmed/16448051
nPYc-Toolbox	Sands et al.[22]	https://www.ncbi.nlm.nih.gov/pubmed/31350543
peakpantheR	Wolfer et al.[23]	https://www.ncbi.nlm.nih.gov/pubmed/34125879

RESOURCE AVAILABILITY

Lead contact

Further information and requests for resources and reagents should be directed to and will be fulfilled by the lead contact, Professor Shiranee Sriskandan (s.sriskandan@imperial.ac.uk).

Materials availability

This study did not generate new unique reagents.

Data code and availability

- Metabolomic and transcriptomic data have been deposited at the European Bioinformatics Institute (EMBL-EBI) MetaboLights repository and are publicly available as of the date of publication. Accession numbers are listed in the key resources table.
- This paper does not report original code.
- Any additional information required to reanalyse the data reported in this paper is available from the lead contact upon request.

EXPERIMENTAL MODEL AND SUBJECT DETAILS

Study design and population

Adult patients presenting to the emergency department were recruited into two separate cohorts: the discovery cohort and a post-hoc external validation cohort.

In the discovery cohort, patient serum samples were obtained from two parallel studies at Imperial College Healthcare NHS Trust (ICHNT), the Imperial arm of the cross-site Bioresource for Adult Infectious Diseases (BioAID),[24] from 15th September 2014 – 4th December 2020 and the Imperial Microbial Products in Infection study, from 18th March 2020 – 4th December 2020. ICHNT patients were admitted via one of two different emergency departments. In the validation cohort, patient serum

samples were obtained from the University College London Hospital (UCLH) Trust arm of BioAID from 30th April 2014 – 20th July 2021.

In both discovery and validation cohorts, patients were recruited to BioAID if they had a suspected clinical infection syndrome of sufficient severity, as assessed by a clinician, to warrant blood culture testing. Blood samples were obtained at the point of admission, alongside microbial isolates identified during the inpatient stay, in conjunction with demographic and clinical data. Ethical approval was obtained to take deferred consent from patients (or next of kin/nominated consultee) to retain blood samples, including serum and RNA specimens, as well as clinical data (South Central – Oxford C Research Ethics Committee [REC] references 14/SC/0008 and 19/SC/0116).[24]

In the discovery cohort, patients were retrospectively identified as part of the Microbial Products in Infection protocol where a pathogen of interest – in this study, SARS-CoV-2 – was identified to the research team by the routine diagnostic laboratory. Serum samples obtained at the point of admission were linked to anonymised patient data provided by an NHS clinician including age, sex, timing of sample in relation to illness onset, survival/death, ICU admission, duration of stay, and blood test results. (West London REC reference 06/Q0406/20). Demographics for all patients are reported in Table S1.

In both BioAID and Microbial Products in Infection, all serum samples were taken in the same manner at the point of patient admission to hospital, prior to any intervention, as part of usual clinical care. Following routine diagnostic testing, surplus volumes were retrieved from the diagnostic laboratory where they had been stored at 4 °C, and transferred to a −80 °C freezer within five days of sample acquisition. During the peaks of the UK COVID-19 pandemic, owing to unprecedented pressures placed on laboratory staffing, transfer to −80 °C of serum samples from n = 80 COVID-19 patients was delayed to between six and 30 days from sample acquisition. These samples were classified into a separate sub-group and were not used in the discovery primary analysis to avoid confounding bias. Control serum samples from consenting healthy donors were from an approved subcollection (MED_SS_12_023) of the Imperial College Healthcare NHS Trust Biomedical Research Council (ICHT BRC) Tissue Bank (Wales REC3 17/WA/0161).

Discovery cohort patient selection

Serum samples from patients in one of the following six categories were used in the discovery cohort: culture-confirmed Gram-positive bacteraemia, culture-confirmed Gram-negative bacteraemia, PCR-confirmed viral infection prior to detection of SARS-CoV-2 in the UK (January 2020), PCR-confirmed COVID-19, non-infected patients, and healthy controls.

N = 24 samples in each of two comparator groups were required to achieve a power of >90% to identify an AUC of at least 0.8, at a significance level of 0.01. Thus to enable all comparisons, accounting for potential sample exclusion (e.g. assay failure, poor data quality), we used n = 30 samples in each clinical group apart from COVID-19, where we included all available samples (n = 112) to facilitate exploration of severity differences in this cohort. Infection categories were assigned using electronic diagnostic pathology data pertaining to admission only and confirmed by a clinician. Non-infected patients were identified from the database where there was no positive microbial diagnostic test and no infection-related ICD-10 diagnostic code from BioAID admission. Sera from n = 13 healthy controls were available from a subcollection of the ICHT BRC Tissue Bank.

To facilitate multi-omic comparison, serum samples from BioAID patients were prioritised if whole blood RNA-Sequencing (RNA-Seq) had already been undertaken as part of an earlier study, where samples had been selected from the BioAID database using a random number generator.[25] Sera from additional BioAID patients were selected randomly from within individual infection groups using a random number generator in Excel.

Bacteraemic patients were excluded if the isolated bacterium was deemed a contaminant, or if the blood culture was taken >24 hours prior to/post the admission serum sample. COVID-19 patients were excluded if their positive PCR test was taken >10 days prior to admission, or >2 days post admission, to avoid non-COVID-19 related admissions and hospital-acquired COVID-19, respectively. Microbiologically confirmed co-infections across different infection classes were excluded.

Validation cohort patient selection

In the validation cohort, we compared two patient groups: bacteraemic and viral. Based on discovery data, to achieve a power of >90% to identify an AUC of 0.75, at a significance level of 0.01, we required 36 patients in each group. Accounting for potential sample exclusion, we used n = 40 samples in each group, selected from the UCLH BioAID database using a random number generator in Excel. For all patients, the same inclusion and exclusion criteria were applied as in the discovery cohort.

METHOD DETAILS

Metabolic profiling assays

Serum samples from 245 patients in the discovery cohort and 80 patients in the validation cohort were analysed using ultra-performance LC-MS following previously described analytical and quality control (QC) procedures.[26] A suite of chromatographic separations was used in the discovery cohort, each coupled with high resolution time of flight mass spectrometry, to maximise coverage of a broad range of metabolite and lipid classes. Small molecule profiling was performed using hydrophilic interaction liquid chromatography (HILIC), while lipid profiling was performed using reversed-phase chromatography (RPC).[27] Each RPC LC-MS assay was conducted in both negative and positive ionisation modes, producing lipid RPC- and lipid RPC + datasets. The HILIC LC-MS assay was conducted in the positive ionisation mode only, producing the HILIC + dataset. In the validation cohort, only the HILIC + assay was undertaken. The final datasets contained the following number of variables; lipid RPC-: 521; lipid RPC+: 2257; HILIC+: 1572 (discovery), 1194 (validation).

Sample preparation and data pre-processing for metabolic profiling

Serum samples were prepared as previously described.[27] Briefly, for each assay, samples were analysed in a randomised order demonstrating no correlation with study design variables, precluding any confounding effect of analysis order. To facilitate quality assessment and pre-processing, a pooled QC sample was prepared by combining equal parts of each study sample and analysed periodically among study sample analyses. In addition, for assessment of analyte response,[28] a series of QC sample dilutions was created (10 × 100%, 5 × 80%, 3 × 60%, 3 × 40%, 5 × 20%, 10 × 10%, 10 × 1%) and analysed at the start and end of each set of sample analyses.

Aliquots (50 μL) were taken from each sample and the pooled QC and diluted 1:1 v/v with ultrapure water. Protein was removed by addition of organic solvent (diluted sample/isopropanol in 1:4 v/v ratio for lipid RPC profiling and diluted sample/acetonitrile in 1:3 v/v ratio for HILIC profiling). Mixtures of method-specific authentic

chemical standards were added at the dilution step (for the HILIC assay) or the protein precipitation step (for the lipid RPC assays) in order to monitor data quality during acquisition. Sample analyses were performed on ACQUITY UPLC instruments (Waters Corp., Milford, MA, USA) coupled to Xevo G2-S Q-TOF mass spectrometers (Waters Corp., Manchester, UK) via a Z-spray electrospray ionisation (ESI) source operating in either positive or negative ion mode.

Raw data were converted to the mzML open-source format and signals below an absolute intensity threshold of 100 counts were removed using the MSConvert tool in ProteoWizard[20] before data extraction using XCMS,[21] outputting a matrix of measurements (peak integrals) organised row-wise into samples and column-wise into LC-MS "features", each of which is described by its mass:charge (*m/z*) value and chromatographic retention time. All datasets were further processed using the nPYc-Toolbox[22] for elimination of potential run-order effects and filtering of features not meeting previously established QC criteria. Only features measured with high analytical quality (RSD in pooled QC<30%, pooled QC dilution series Pearson correlation to dilution factor>0.7, RSD in study samples>1.1* RSD in pooled QC) were retained and put forward for further statistical analysis.

Metabolite identification

An in-house R-script was used to collate all LC-MS features in the discovery cohort with peak elution profiles (extracted ion chromatograms) highly correlated to that of the feature identified as a discriminator for viral infections (*m/z* of 248.0647 and retention time of 1.96 mins), revealing the putative mass spectrum of the metabolite biomarker comprising five features. The original feature *m/z* 248.0647 was assigned as the $[M+Na]^+$ ion species, and the four associated features were assigned as the first isotope of $[M+Na]^+$ ion at *m/z* 249.0676, $[M+H]^+$ at *m/z* 226.0827, $[M+K]^+$ at *m/z* 264.0383, and an in-source fragment at *m/z* 112.0517. Targeted extraction of the four associated features, each absent from the XCMS profiling dataset owing to non-detection, was performed using peakPantheR software and pre-processed as described above for the XCMS profiling dataset.[23] Each associated feature gave comparable individual AUC to the $[M+Na]^+$ ion species identified from analysis of the profiling data (AUC 0.954) when comparing viral versus all other groups (Methods Table S1).

The molecular formula of the metabolite of interest was determined to be C9H11N3O4 by elemental composition of the [M+H]+ ion species utilising accurate mass and observed isotope distribution. Tandem mass spectrometry (MS/MS) utilising collision induced dissociation (CID) was performed on both the $[M+H]^+$ and $[M+Na]^+$ ion species, revealing the fragmentation patterns illustrated in Methods Figure S1.

The experimental MS/MS spectrum for the $[M+H]^+$ ion species (*m/z* 226.0827) revealed the loss of one and two water moieties (*m/z* 208.072 and *m/z* 190.062, respectively) and the fragment with *m/z* 112.05 consistent with cytosine ($[M+H]^+$). The experimental MS/MS spectrum for the $[M+Na]^+$ ion species (*m/z* 248.0647) revealed analogous species.

No matches were found for these spectra in databases (Human Metabolome Database,[29] METLIN,[30] NIST17,[31] Mass Bank of North America [http://massbank.us/]). However, the apparent presence of a cytosine fragment led to the hypothesis that the metabolite of interest was a nucleoside previously reported in the literature – 3′-Deoxy-3′,4′-didehydro-cytidine (ddhC), a free base of the antiviral

ribonucleotide 3′-Deoxy-3′,4′-didehydro-cytidine triphosphate.[12] The experimental MS/MS spectrum of $[M+H]^+$ obtained in our study samples matched the previously published fragmentation pattern of natural ddhC detected in cell lysates of *E. coli* and authentic chemical standard of ddhC.[13] The HILIC method used for MS/MS analysis was the same as that used in the profiling method with the same MS source conditions.[26] MS/MS target selection was performed using unit mass selection via quadrupole with a collision energy voltage ramp of 10-45V.

Finally, the structure of metabolite was definitively identified by analysing a chemical reference standard of ddhC (acquired from Berry & Associates) in parallel with the study samples, using the HILIC profiling method and MS/MS to match chromatographic retention time and fragmentation spectrum, respectively (Methods Figure S2). For the former, a spike-in technique was used, where the pooled serum sample was mixed with different concentrations of the ddhC chemical standard (2.5, 5 and 10 ng/mL).

For the validation cohort, the identification of ddhC was based on the same *m/z* and similar retention time to that observed in the discovery data.

Multi-omic comparison

We examined the interaction between whole blood gene expression and the feature of interest identified in the discovery cohort. Gene expression data were obtained from RNA-Seq of Imperial BioAID patient RNA samples, performed prior to this study in two cohorts. Full details for the first patient cohort (recruited pre-COVID-19 pandemic) have been described previously.[25] For the second patient cohort (recruited during the COVID-19 pandemic), whole blood was collected in the same way as the first cohort.[25] Material was quantified using RiboGreen (Invitrogen) on the FLUOstar OPTIMA plate reader (BMG Labtech) and the size profile and integrity analysed on the 2200 TapeStation (Agilent, RNA ScreenTape). Input material was normalised and strand specific library preparation was completed using NEBNext Ultra II mRNA kit (NEB) and NEB rRNA/globin depletion probes following manufacturer's instructions. Libraries were on a Tetrad (Bio-Rad) using in-house unique dual indexing primers (based on Lamble et al.).[32] Individual libraries were normalised using Qubit and pooled together. The pooled library was diluted to ~10 nM for storage and denatured and further diluted prior to loading on the sequencer. Paired end sequencing was performed The Wellcome Centre for Human Genetics in Oxford UK using a Novaseq6000 platform at 150 paired end configuration, generating a raw read count of 30 million reads per sample. The RNA-Seq analysis pipeline consisted of quality control using FastQC,[33] MultiQC[34] and annotations modified with BEDTools,[35] alignment and read counting using STAR,[36] SAMtools,[37] FeatureCounts[38] and version 89 ensembl GCh38 genome and annotation.[39]

Genes completely missing in either of the RNA-Seq cohorts were removed, in addition to ribosomal genes. The two RNA-Seq cohorts were merged and the batch effects between the two cohorts, in addition to the plate effects within the first cohort, were removed by combat_seq.[40] The raw counts were normalised using DESeq2.[41] In patients for whom both metabolic and transcriptomic data were available, we assessed the correlation between $\log_2$-transformed feature intensities of a metabolite of interest and $\log_2$-transformed expression of associated genes using Pearson correlation coefficients. We restricted further analysis to the five genes most highly correlated to the metabolite of interest.

QUANTIFICATION AND STATISTICAL ANALYSIS

Data analysis was performed using R.[42] Power calculations were performed using the pROC package.[19] Unit-variance scaled principal component analysis (PCA) and eigencor plots were performed to identify the major sources of variation in the datasets, using the PCAtools package.[18] For PCA, features where >98.5% of samples returned an intensity of zero were excluded (n = 3/1572 in discovery HILIC + dataset, nil in both discovery lipidomics datasets, n = 1/1194 in validation HILIC + dataset).

In the discovery cohort, we compared all viral cases (COVID-19 and pre-COVID-19) versus others, all bacterial cases (Gram-positive and Gram-negative bacteraemia) versus others, and all viral versus all bacterial cases. In the validation cohort, we compared all viral cases versus all bacterial cases. In each comparison, we assessed the fold-change between the infection groups' median intensities for each feature. P-values were generated using the two-sided Wilcoxon test and were adjusted using the Benjamini-Hochberg procedure.[43] Volcano plots were generated comparing median $\log_2$fold-change and -$\log_{10}$ p-values.

In order to cross-validate findings in the discovery cohort, we used the variable selection method, forward selection-partial least squares (FS-PLS).[17] FS-PLS has been described in detail elsewhere.[7,44] Briefly, it is a forward-selection method that selects variables most strongly associated with the groups of interest. It can be used to select a multi-feature signature composed of non-correlated variables, but in this study the 'max' parameter was set to one to evaluate the performance with only one feature. Feature intensities were $\log_2$ transformed. A p-value threshold of 0.01 was used, which determined the selection of a variable or termination. 100 runs of FS-PLS were applied to the dataset for every comparison, each time with a different training:test split at a ratio of 70:30. In each FS-PLS run, the feature identified on the training set was tested on the test set, and its performance was assessed using the AUC generated. For the feature that was selected in the most FS-PLS runs out of 100, the median and interquartile range (IQR) of the respective test AUCs were generated.

To assess the diagnostic utility of a feature of interest in the discovery cohort and compare it to the traditional biomarkers C-reactive protein (CRP), white cell count, and lymphocyte count (procalcitonin levels were not routinely available), as well as examine its use in the validation cohort, AUCs were generated using the pROC package.[19] The Youden's J statistic was used to determine thresholds for sensitivity and specificity.[45]

In an exploratory post-hoc analysis, to investigate the relationship between the intensity of a feature of interest and illness severity in viral infections in the discovery cohort, we developed a three-point severity scale that differentiated between mild, moderate and severe illness in both COVID-19 and other viral illnesses. We incorporated duration of hospital admission, re-admission to hospital, admission to the intensive care unit (ICU) and death in this scale. Severity group 1 (mild) included patients admitted to hospital for 0–2 days, group 2 (moderate) included patients admitted for 3–8 days, and group 3 (severe) included patients admitted for >8 days and those who were admitted to ICU or died at any point during admission. Re-admission to hospital within 5 days of discharge was counted as the same admission. P-values were generated using the Kruskal-Wallis test.

Med, Volume *3*

Supplemental information

Antiviral metabolite 3′-deoxy-3′,4′-didehydro-cytidine is detectable in serum and identifies acute viral infections including COVID-19

Ravi Mehta, Elena Chekmeneva, Heather Jackson, Caroline Sands, Ewurabena Mills, Dominique Arancon, Ho Kwong Li, Paul Arkell, Timothy M. Rawson, Robert Hammond, Maisarah Amran, Anna Haber, Graham S. Cooke, Mahdad Noursadeghi, Myrsini Kaforou, Matthew R. Lewis, Zoltan Takats, and Shiranee Sriskandan

Antiviral metabolite 3'-Deoxy-3',4'-didehydro-cytidine is detectable in serum and identifies acute viral infections including COVID-19

Supplementary Information

Supplementary Table 1 (related to Figure 1). Patient demographics and sample numbers in discovery and validation cohorts. 2

Supplementary Table 2 (related to Figure 1). Confirmed pathogens in discovery and validation cohorts. 3

Supplementary Table 3 (related to Figure 2). Cross-validation of feature performance using FS-PLS. 4

Supplementary Table 4 (related to Figure 4). Five gene transcipts most highly correlated with ddhC in the discovery primary analysis cohort. 5

Supplementary Figure 1 (related to Figure 2). Discovery cohort metabolomic findings and discovery of ddhC. 6

Supplementary Figure 2 (related to Figure 2). Discovery cohort AUCs and relative feature intensity data for all samples and influence of host demographic and clinical factors on feature intensity. 8

Supplementary Figure 3 (related to Figure 3). Validation cohort metabolomic findings and severity analysis. 10

Supplementary Figure 4 (related to Figure 4). Correlation between ddhC and CMPK2 expression in different patient groups in the discovery cohort. 12

Methods S1 – Supplementary items related to STAR Methods metabolite identification (separate file)

Table 1 *Targeted feature extraction related to STAR Methods metabolite identification*

Figure 1 *Tandem mass spectrometry related to STAR Methods metabolite identification*

Figure 2 *Definitive identification of ddhC using a chemical standard related to STAR Methods metabolite identification*

Supplementary Table 1 (related to Figure 1). Patient demographics and sample numbers in discovery and validation cohorts.

DISCOVERY COHORT	Gram +ve bacteraemia	Gram -ve bacteraemia	Pre-COVID-19 viral	COVID-19	Non-infected unwell control	Healthy control	Total
**n (included in at least one assay)**	30	30	29	32	30	13	164
**n (lipid RPC-)**	30	30	29	31	30	13	163
**n (lipid RPC+)**	29	30	29	31	29	12	160
**n (HILIC+)**	29	30	29	31	29	13	161
**Age (median [IQR])**	71 [59-80]	69 [60-79]	41 [26-54]	66 [60-80]	52 [43-68]	34 [31-45]	60 [44-74]
**Female (n [%])**	18 [60]	14 [47]	14 [48]	18 [56]	12 [40]	8 [62]	84 [51]
**WCC x10^9/L (median [IQR])**	11.6 [7.3-14.8]	13.5 [8.9-16.4]	7.8 [5.1-9.5]	7.2 [5.3-9.4]	8.8 [6.6-12.2]	NA	8.9 [6.2-12.9]
**Lymphocyte count x10^9/L (median [IQR])**	0.9 [0.5-1.3]	0.7 [0.5-1.2]	0.6 [0.8-1.0]	1.0 [0.9-1.3]	1.8 [1.0-2.7]	NA	0.9 [0.6-1.5]
**CRP mg/L (median [IQR])**	56 [27-107]	137 [42-205]	31 [17-74]	89 [50-154]	6 [1-15]	NA	56 [15-120]
**VALIDATION COHORT**	**Bacteraemia**		**Viral**		**NA**	**NA**	**Total**
**n (HILIC+)**	40		40		-	-	80
**Age (median [IQR])**	66 [52-77]		55 [45-72]		-	-	61 [48-73]
**Female (n [%])**	23 [58]		20 [50]		-	-	43 [54]

Supplementary Table 1 (related to Figure 1). Patient demographics and sample numbers in discovery and validation cohorts. HILIC = hydrophilic interaction chromatography, RPC = reversed-phase chromatography, IQR = interquartile range, WCC = white cell count, CRP = C-reactive protein, NA = not applicable. In the discovery cohort, routine haematology and biochemistry (WCC, lymphocyte count & CRP) were not available for the 13 healthy controls; CRP values based on 125/151 patients.

Supplementary Table 2 (related to Figure 1). Confirmed pathogens in discovery and validation cohorts.

	DISCOVERY						VALIDATION	
**Patient sub-group**	**Gram +ve bacteraemia**	**Gram -ve bacteraemia**	**Pre-COVID-19 viral**	**COVID-19**	**Non-infected unwell**	**Healthy control**	**Bacterial**	**Viral**
**n (total)**	30	30	29	32	30	13	40	40
Staphylococcus aureus	10	-	-	-	-	-	7	-
Alpha-haemolytic Streptococcus (viridans)	5	-	-	-	-	-	1	-
Streptococcus pneumoniae	4	-	-	-	-	-	2	-
Beta-haemolytic Streptococcus	4	-	-	-	-	-	4	-
Enterococcus faecalis	3	-	-	-	-	-	-	-
Enterococcus casseliflavus	1	-	-	-	-	-	-	-
Listeria monocytogenes	1	-	-	-	-	-	-	-
Clostridium septicum	1	-	-	-	-	-	-	-
Lactobacillus paracasei	1	-	-	-	-	-	-	-
Escherichia coli	-	19	-	-	-	-	21	-
Klebsiella sp.	-	4	-	-	-	-	-	-
Proteus mirabilis	-	2	-	-	-	-	-	-
Enterobacter cloacae	-	2	-	-	-	-	1	-
Serratia marcescens	-	1	-	-	-	-	-	-
Moraxella catarrhalis	-	1	-	-	-	-	-	-
Haemophilus parainfluenzae	-	1	-	-	-	-	-	-
Pseudomonas aeruginosa	-	-	-	-	-	-	2	-
Salmonella typhi	-	-	-	-	-	-	1	-
Acinetobacter junii	-	-	-	-	-	-	1	-
**Influenza A**	-	-	11	-	-	-	-	7
**Adenovirus**	-	-	5	-	-	-	-	1
**Measles**	-	-	3	-	-	-	-	-
**Varicella Zoster Virus**	-	-	3	-	-	-	-	-
**Dengue**	-	-	2	-	-	-	-	-
**Herpes Simplex Virus**	-	-	1	-	-	-	-	3
**Rhinovirus / Adenovirus / Parainf 1***	-	-	1	-	-	-	-	-
**Parainfluenza 2**	-	-	1	-	-	-	-	-
**Herpes Simplex Virus + Adenovirus**	-	-	1	-	-	-	-	-
**Enterovirus**	-	-	1	-	-	-	-	-
**Rhinovirus**	-	-	-	-	-	-	-	6
**Influenza B**	-	-	-	-	-	-	-	5
**Metapneumovirus**	-	-	-	-	-	-	-	2
**Norovirus**	-	-	-	-	-	-	-	1
**Rotavirus**	-	-	-	-	-	-	-	1
**Respiratory Syncytial Virus**	-	-	-	-	-	-	-	1
**Influenza A + Rhinovirus**	-	-	-	-	-	-	-	1
**SARS-CoV-2**	-	-	-	32	-	-		12

Supplementary Table 2 (related to Figure 1). Confirmed pathogens in discovery and validation cohorts. PCR (viral)- or culture (bacterial)-confirmed pathogens in the discovery primary analysis (n=164) and validation (n=80) cohorts. *All three of rhinovirus, parainfluenza 1 virus and adenovirus (weak positive) were detected on a respiratory viral PCR assay for this patient.

Supplementary Table 3 (related to Figure 2). Cross-validation of feature performance using FS-PLS.

	Assay	Most frequent feature	FS-PLS runs (/100)	Median test AUC	IQR test AUC
**Viral vs. other**	Lipid RPC+	778.54/5.01	61	0.843	0.809-0.867
	Lipid RPC-	766.53/5.76	46	0.775	0.724-0.802
	HILIC+	248.06/1.96	100	0.957	0.943-0.970
**Viral vs. bacterial**	Lipid RPC+	711.58/5.90	37	0.833	0.796-0.867
	Lipid RPC-	717.53/6.00	39	0.731	0.701-0.779
	HILIC+	248.06/1.96	99	0.951	0.926-0.969
**Bacterial vs. other**	Lipid RPC+	740.61/6.62	31	0.741	0.710-0.794
	Lipid RPC-	871.69/8.09	30	0.768	0.726-0.813
	HILIC+	512.34/5.09	69	0.797	0.767-0.839

Supplementary Table 3 (related to Figure 2). Cross-validation of feature performance using FS-PLS.
Cross-validation of feature performance in differentiating viral versus other, bacteraemic versus viral and bacteraemic versus other using 100 forward selection-partial least squares (FS-PLS) runs comprising 70:30 training:test splits. FS-PLS runs (/100) = the number of times the feature was selected in 100 FS-PLS runs. ddhC (248.06/1.96) was selected as the discriminating feature in all 100 FS-PLS runs in the HILIC+ assay comparing viral versus other, generating a median test area under the receiver operating characteristic curve (AUC) of 0.957. Features are shown by *m/z*/retention time. IQR = interquartile range, HILIC = hydrophilic interaction chromatography, RPC = reversed-phase chromatography.

Supplementary Table 4 (related to Figure 4). Five gene transcripts most highly correlated with ddhC in the discovery primary analysis cohort.

Gene	Ensembl ID	Correlation with ddhC	p-value
CMPK2	ENSG00000134326	0.763	$< 1x10^{23}$
SPATS2L	ENSG00000196141	0.759	$< 1x10^{23}$
IFI27	ENSG00000165949	0.756	$< 1x10^{23}$
RSAD2 (viperin)	ENSG00000134321	0.748	$< 1x10^{22}$
Unnamed*	ENSG00000233785	0.743	$< 1x10^{21}$

Supplementary Table 4 (related to Figure 4). Five gene transcripts most highly correlated with ddhC in the discovery primary analysis cohort. Pearson's correlation coefficients between log_2-transformed whole blood-derived gene expression and log_2-transformed ddhC intensity, with the five (of 18,248) most highly correlated genes shown (n=122 patients). *No identification available.

Supplementary Figure 1 (related to Figure 2). Discovery cohort metabolomic findings and discovery of ddhC.

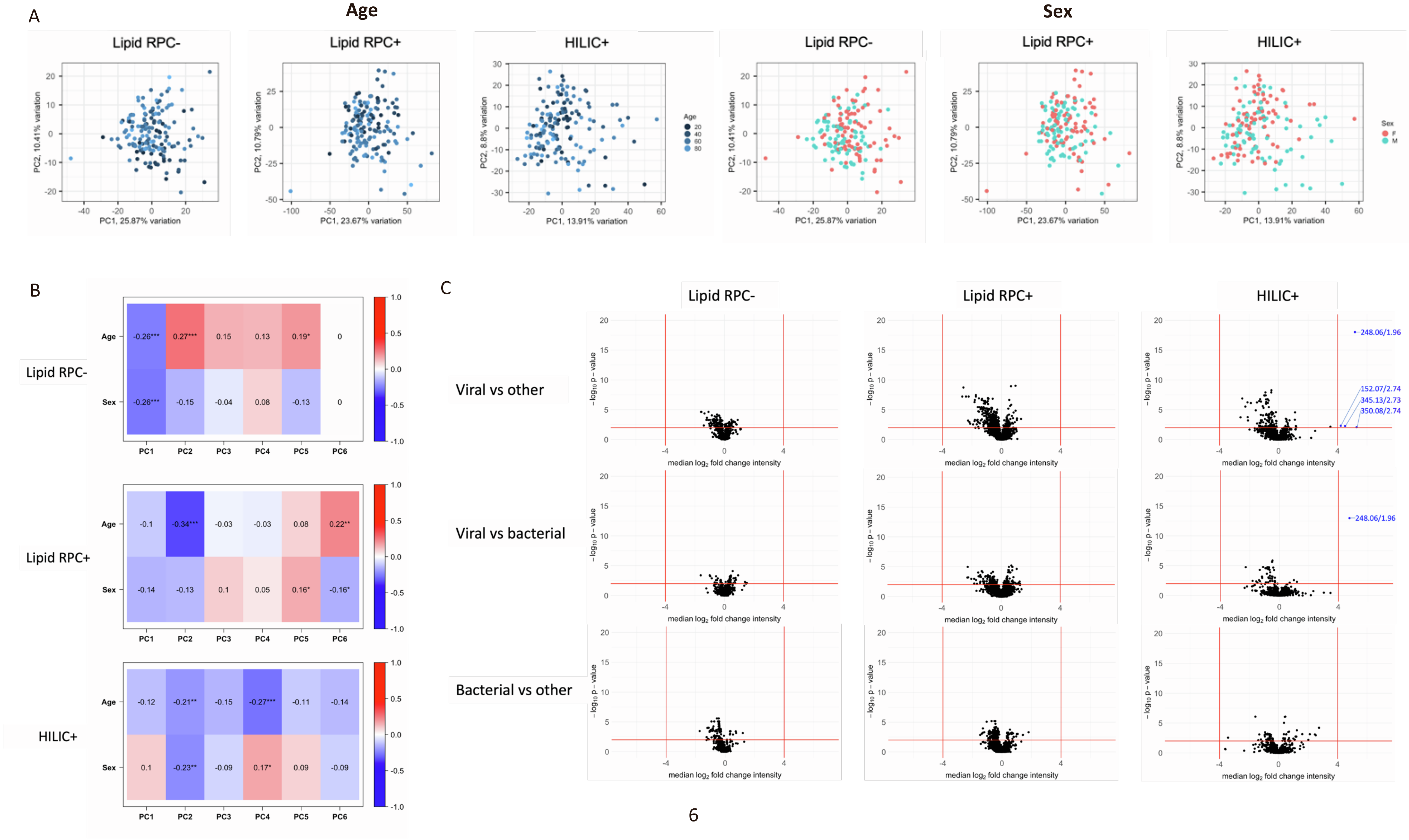

Supplementary Figure 1 (related to Figure 2). Discovery cohort metabolomic findings and discovery of ddhC.

A. Principal component analysis of lipid profiling (lipid RPC-, lipid RPC+) and small molecule profiling (HILIC+) data from discovery primary analysis cohort, showing no variance due to age or sex (n=161-163 samples).

B. Eigencor plots show minimal correlation between principal component variance and age or sex in discovery primary analysis cohort (n=161-163). ***p-value<0.001, **<0.01, *<0.05.

C. Volcano plot showing median $\log_2$ fold change intensity of all features versus $-\log_{10}$ p-value in all assays when comparing: viral versus other, viral versus bacterial, and bacterial versus other groups in discovery primary analysis cohort (n=161-163). Threshold lines in red represent a fold-change of 16 [$\log_2$(foldchange) of 4] and p-value of 0.01 [$-\log_{10}$(p-value) of 2]. Candidate biomarkers are shown in blue by their *m/z*/retention time. P-values generated using the two-sided Wilcoxon test and adjusted using the Benjamini-Hochberg procedure.

HILIC = hydrophilic interaction chromatography, RPC = reversed-phase chromatography.

Supplementary Figure 2 (related to Figure 2). Discovery cohort AUCs and relative feature intensity data for all samples and influence of host demographic and clinical factors on feature intensity.

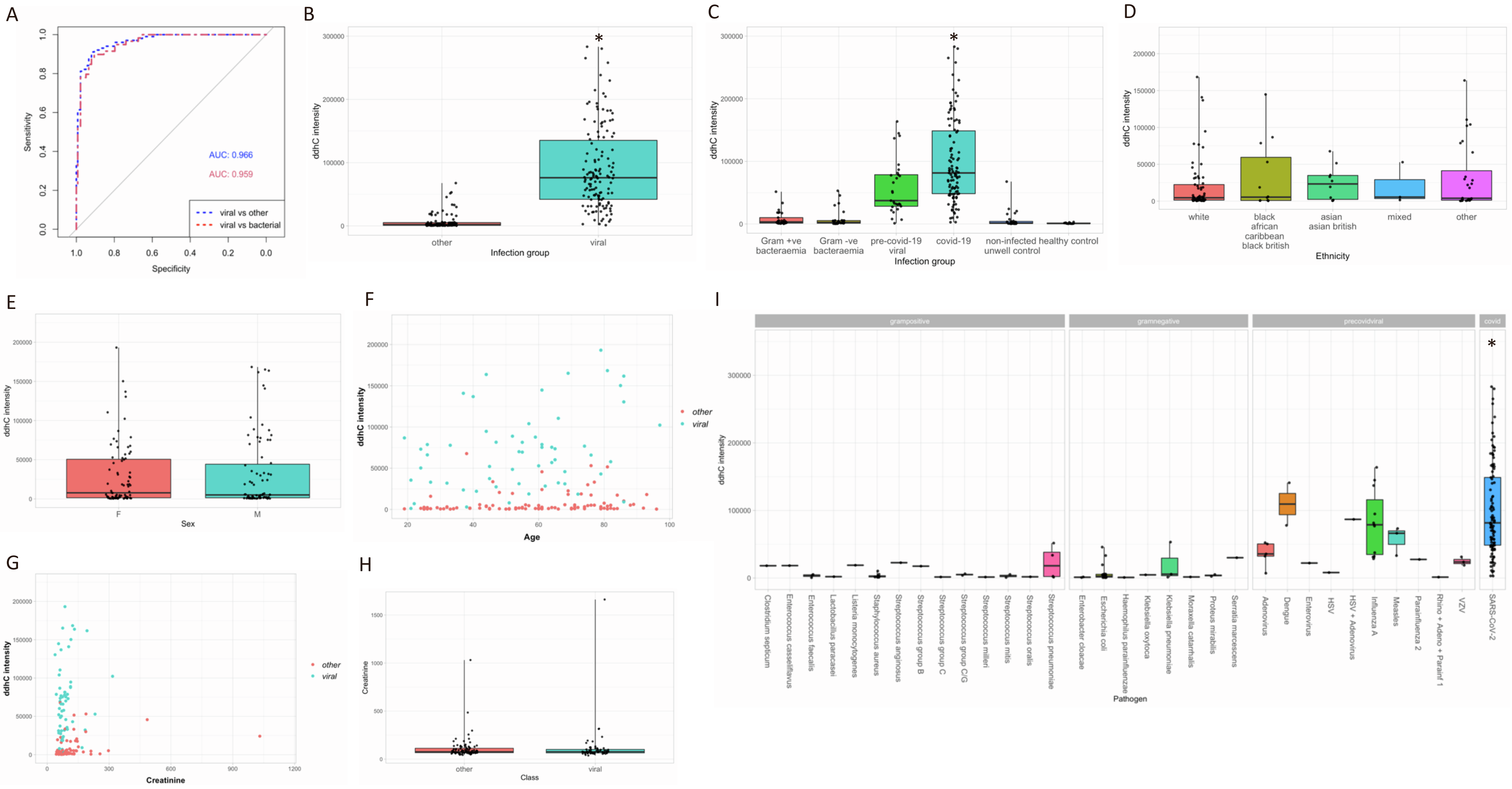

Supplementary Figure 2 (related to Figure 2). Discovery cohort AUCs and relative feature intensity data for all samples and influence of host demographic and clinical factors on feature intensity.
For all plots, ddhC feature *(m/z*/retention time of 248.06/1.96) intensity was used.

A. AUCs for ddhC distinguishing viral (pre-COVID-19 viral and COVID-19) versus all other and viral versus bacterial (Gram-positive and Gram-negative bacteraemia) groups using all discovery cohort samples, including those that spent >5days outside a -80°C freezer. **Blue.** AUC of 0.966 for ddhC differentiating viral infections from all other groups (n=239). **Red.** AUC of 0.959 for ddhC differentiating viral from bacterial infection, with controls omitted (n=197).

B & C. ddhC intensity data in different patient groups using all discovery cohort samples, including those that spent >5days outside a -80°C freezer (n=239). **B.** Viral versus all other groups. **C.** All comparator groups. *4 samples in the COVID-19 group had an intensity of >400000, not shown.

D-H. Effect of ethnicity, sex, age, and renal function on ddhC intensity in the discovery primary analysis cohort. No significant difference in median ddhC intensity between **D.** ethnicity groups (n=123 with available ethnicity data) or **E.** sex (n=161), p-value threshold <0.01. **F.** No significant correlation between ddhC intensity and age in the discovery primary analysis cohort (n=161). **G.** Low correlation between ddhC intensity and serum creatinine (n=148, healthy controls not included as creatinine not available, Pearson correlation coefficient 0.555, p-value $<1x10^{-12}$). **H.** No significant difference in median creatinine between viral and other groups (n=148), p-value threshold <0.01. 2 samples in the COVID-19 group had a relative intensity of >700000, not shown in any D-H plots. P-values for median comparisons generated using the two-sided Wilcoxon test (E, H) and Kruskal-Wallis test (D).

I. Effect of causative pathogen on serum ddhC intensity in the total discovery cohort. Points represent individual patients (including samples that spent >5days outside a -80°C freezer, n=239). *4 samples in the COVID-19 group had an intensity of >400000, not shown.

Scatter plots: non-viral group (red points) includes bacteraemic patients, non-infected unwell controls, and healthy controls; viral group (blue points) includes COVID-19 and pre-COVID-19 viral infection patients. Box plots: points represent individual patients; boxes represent interquartile ranges with medians.

Supplementary Figure 3 (related to Figure 3). Validation cohort metabolomic findings and severity analysis.

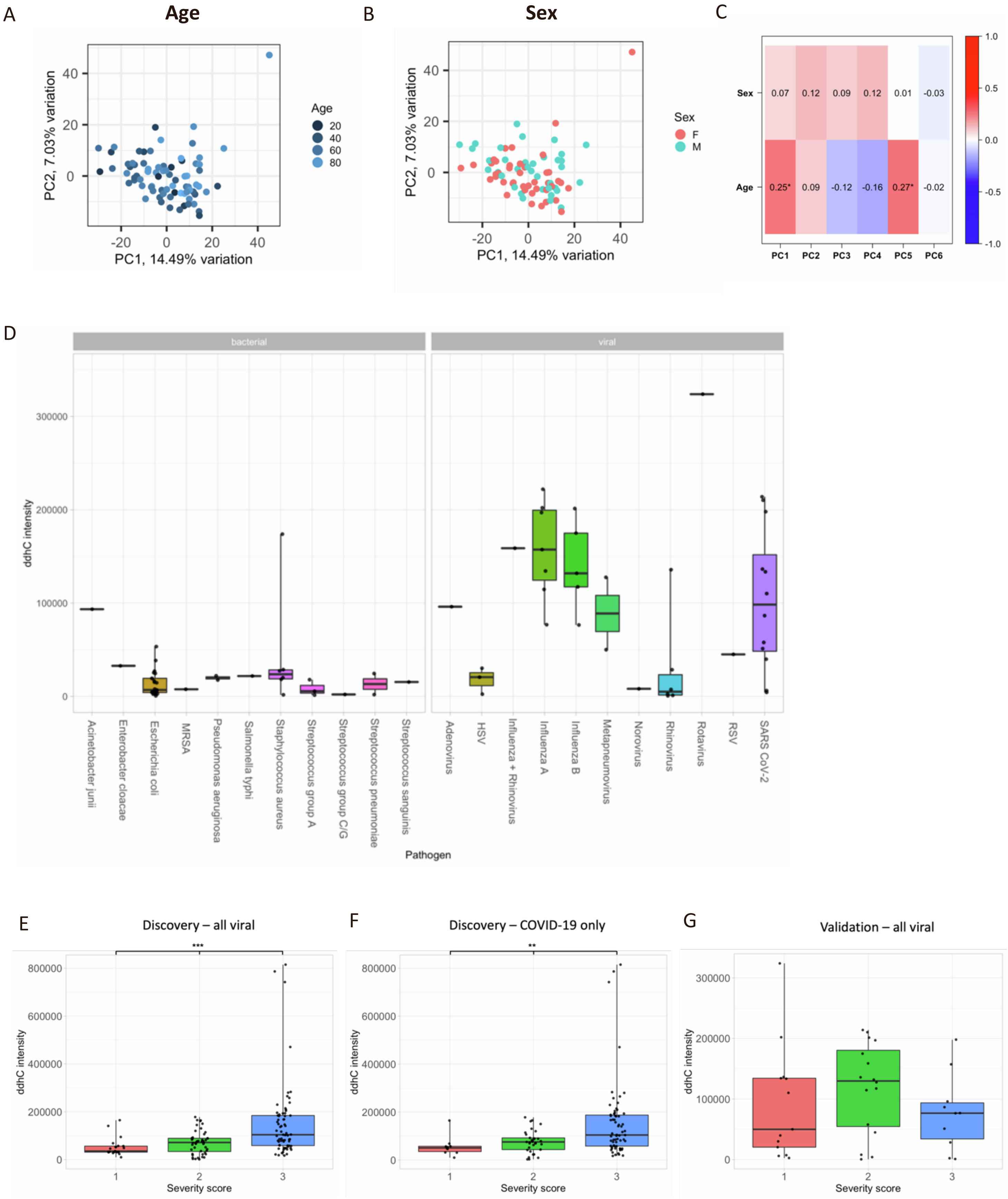

Supplementary Figure 3 (related to Figure 3). Validation cohort metabolomic findings and severity analysis.

A & B. Principal component analysis of small molecule profiling (HILIC+) data from validation cohort, showing no variance due to age or sex (n=80).

C. Eigencor plot shows minimal correlation between principal component variance and age or sex in validation cohort (n=80). *p-value<0.05.

D. ddhC intensity by pathogen type in validation cohort (n=80).

E-G. Severity analysis in discovery and validation cohorts. Association between median ddhC intensity (y axis) and outcome severity in all viral cases (pre-COVID-19 viral and COVID-19) in the total discovery patient cohort (**E**) (n=138), ***p-value $<1x10^{-5}$, and in COVID-19 cases only (**F**) (n=109), in the total discovery patient cohort **p-value $<1x10^{-3}$. **G.** No significant association found between outcome severity and ddhC intensity in all viral cases in the validation cohort (n=40). Severity score: 1 = admission duration 0-2 days, 2 = admission duration 3-8 days, 3 = admission duration >8 days, ICU admission or death. p-values generated using the Kruskal-Wallis test.

Box plots: points represent individual patients; boxes represent interquartile ranges with medians.

Supplementary Figure 4 (related to Figure 4). Correlation between ddhC and *CMPK2* expression in different patient groups in the discovery cohort.

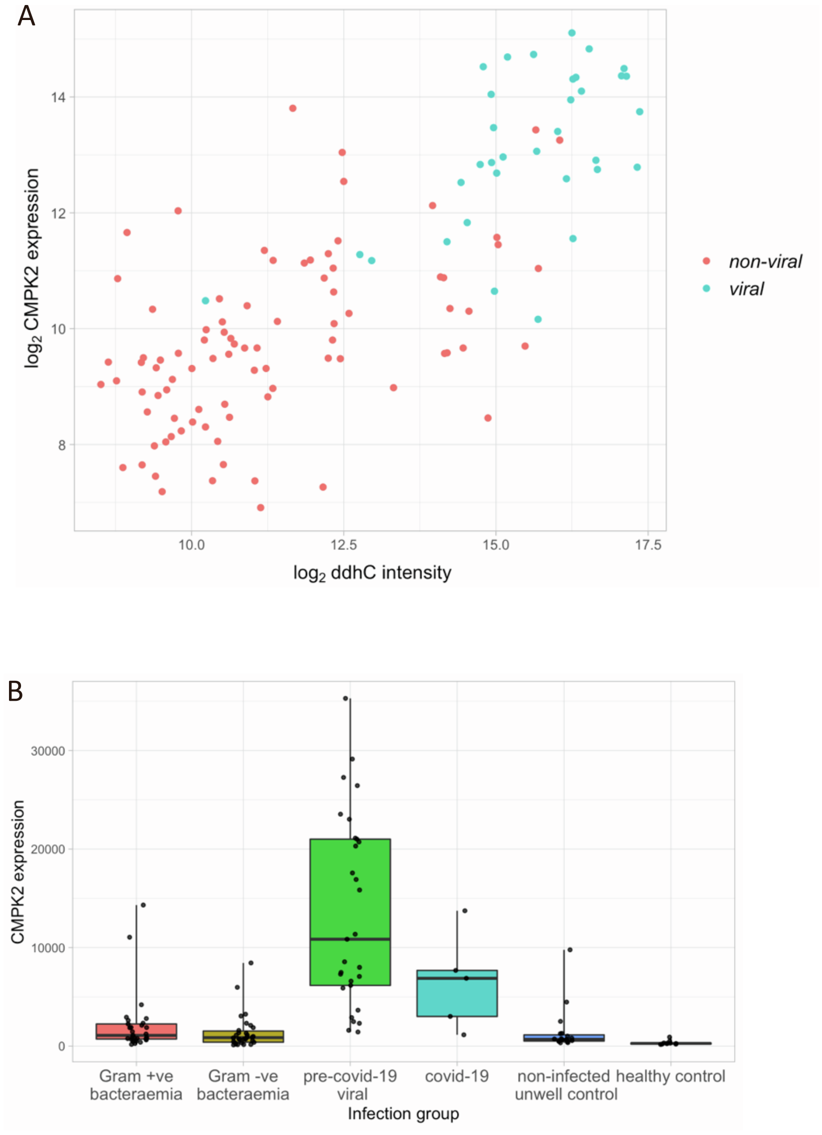

Supplementary Figure 4 (related to Figure 4). Correlation between ddhC intensity and CMPK2 expression in different patient groups in the discovery cohort.

A. Correlation between ddhC intensity and *CMPK2* gene expression in 122 patients in the discovery cohort. Non-viral group (red points) includes bacteraemic patients, non-infected unwell controls, and healthy controls; viral group (blue points) includes COVID-19 and pre-COVID-19 viral infection patients. Pearson correlation coefficient = 0.763, p-value < $1x10^{-23}$.

B. Normalised *CMPK2* gene counts for 122 patients in different infection groups. Points represent individual patients. Boxes represent interquartile ranges with medians.
